# Effect of the Configuration of a Bulky Aluminum Initiator on the Structure of Copolymers of *l*,*l*-Lactide with Symmetric Comonomer Trimethylene Carbonate

**DOI:** 10.3390/polym10010070

**Published:** 2018-01-13

**Authors:** Marta Socka, Ryszard Szymanski, Stanislaw Sosnowski, Andrzej Duda

**Affiliations:** Centre of Molecular and Macromolecular Studies, Polish Academy of Sciences, 90-363 Lodz, Sienkiewicza 112, Poland; stasosno@cbmm.lodz.pl

**Keywords:** biodegradable copolyesters, copolymerization kinetics, copolymer microstructure, simulation, reactivity ratios, dilatometry

## Abstract

The effect of configuration of an asymmetric bulky initiator 2,2′-[1,1′-binaphtyl-2,2′-diyl- bis-(nitrylomethilidyne)]diphenoxy aluminum isopropoxide (**Ini**) on structure of copolymer of asymmetric monomer *l*,*l*-lactide (**Lac**) with symmetric comonomer trimethylene carbonate (**Tmc**) was studied using polarimetry, dilatometry, Size Exclusion Chromatography (SEC), and Carbon Nuclear Magnetic Resonance (^13^C NMR). When the *S*-enantiomer of **Ini** was used the distribution in copolymer chains at the beginning of polymerization is statistical, with alternacy tendency, changing next through a gradient region to homoblocks of **Tmc**. However, when *R*-**Ini** was used, the product formed was a gradient oligoblock one, with **Tmc** blocks prevailing at the beginning, changing to **Lac** blocks dominating at the end part of chains. Initiation of copolymerization with the mixture of both initiator enantiomers (*S*:*R* = 6:94) gave a multiblock copolymer of similar features but shorter blocks. Analysis of copolymerization progress required complex analysis of dilatometric data, assuming different molar volume contraction coefficients for units located in different triads. Comonomer reactivity ratios of studied copolymerizations were determined.

## 1. Introduction

Synthetic biodegradable polymers, e.g., aliphatic polyesters and polycarbonates, as well as copolymers of the corresponding monomers, have attracted increasing attention because of their useful properties for applications in the medical field as materials for temporary medical devices, such as scaffolds in tissue engineering or tissue reconstruction and drug-controlled-delivery systems [1,2].

Particularly, high modulus and high strength polylactides (PLac) have received special interest, as lactide (**Lac**) derives from annually renewable resources (i.e., corn starch or sugarcane). To the currently available products obtained from PLac belong sutures, GTR (guided tissue regeneration), orthopedic implants, and implantable drug delivery systems [3,4,5].

However, due to PLac brittleness and relatively low resistance to oxygen and water vapor permeation, the range of possible applications of polylactides is restricted. Those properties could be altered by incorporation of different, suitable comonomer units into the main chain of PLac.

**Scheme 1:**
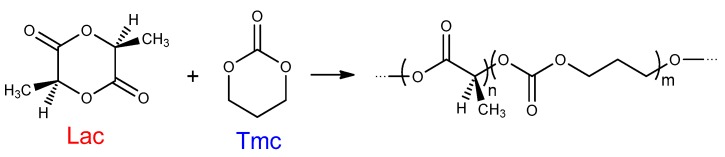
Copolymerization of asymmetric monomer *l*,*l*-lactide (**Lac**) and symmetric comonomer trimethylene carbonate (**Tmc**).

Copolymers containing lactide and carbonate units (e.g., trimethylene carbonate (**Tmc**), Scheme 1):

They are especially interesting due to their increased flexibility and reduced acidity of the degradation products [6].

A ring-opening (co)polymerization (ROP) of aliphatic cyclic esters and carbonates is known as the most convenient method for the controlled synthesis of biodegradable and biocompatible copolymers [7]. Systematic studies on homopolymerization of lactones, lactides, and cyclic carbonates allowed to establish the fundamental thermodynamic, kinetic, and stereochemical aspects of these processes [7,8,9,10]. Particularly, controlled coordination polymerization of lactones and lactides that allowed preparation of polyester from oligomers to high molar mass polymers (*M*_n_ ~ 10^6^) with desired end groups, have been elaborated in our group [11].

In preparation of **Tmc**/**Lac** copolymers the tin derivatives are the most widely used catalyst/initiator systems [12,13,14,15,16,17,18]. The valuable results have also been obtained with various aluminum [19], lanthanide [20,21,22], and zirconium complexes [23]. The homopolymerization rates of **Tmc** and **Lac** are substantially different. Previous studies with the yttrium [24] and calcium [25] complexes proved that the rate of polymerization of **Tmc** is much higher than that for **Lac**. Nevertheless, during copolymerization of **Tmc** and **Lac**, both comonomers possess nearly the same reactivity ratio or the lactide monomer reveals higher reactivity. For example Yasuda et al. [26] have reported the formation of random **Tmc**/**Lac** copolymers in which both monomers exhibited similar reactivity ratios, using SmMe(C_5_Me_5_)_2_THF initiator.

On the other hand, Spassky et al. [24] have reported the formation of almost pure block structure, in process initiated with yttrium alkoxide. The copolymerization of an equimolar mixture of **Lac** and **Tmc** leads to the formation of block copolymers. **Lac** was consumed first due to its significantly higher reactivity ratio. Similarly, the reactivity ratios reported by Dobrzynski [23] (*r*_Lac_ = 13.0 and *r*_Tmc_ = 0.53) proved favorable incorporation of repeating units derived from **Lac** into the copolymer chain. The product *r*_Lac_ × *r*_Tmc_ = 6.89 determined for the copolymerization initiated with zirconium complex was significantly higher than that previously reported for copolymerizations initiated with samarium complex [22] (*r*_Lac_ × *r*_Tmc_ = 1.81, *r*_Lac_ = 7.24, and *r*_Tmc_ = 0.25), and it was the evidence for a strong tendency to form a copolymer with a block structure.

The observed reactivities of **Tmc** and **Lac** in the copolymerization are reversed in comparison with their reactivities in homopolymerization, where the observed rates of polymerization of **Tmc** are higher than of **Lac** while applying the same initiator. Although the first report describing this puzzling phenomenon appeared about twenty years ago [24], to this day there is no plausible explanation on the molecular level.

**Scheme 2:**
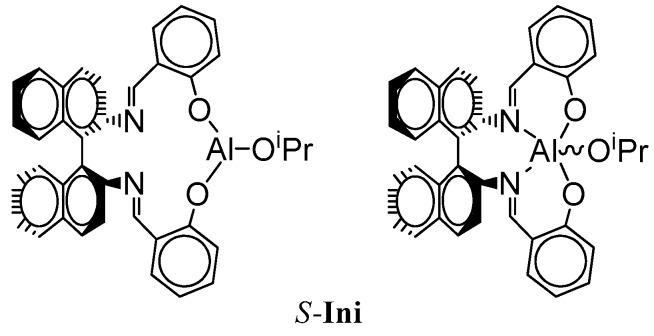
Structure of *S*-2,2′-[1,1′-binaphtyl-2,2′-diyl-bis-(nitrylomethilidyne)]-diphenoxy aluminum isopropoxide (*S*-**Ini**). *R*-**Ini** enantiomer differs with conformation of binaphtyl residue, restricted due to inhibited rotation. Although the pentacoordinated structure is probably dominating in the reaction medium, we, for the sake of simplification, use tricoordinated structures in our other schemes.

The present work shows the preliminary results of our investigation of the effect of configuration of a bulky asymmetric initiator 2,2′-[1,1′-binaphtyl-2,2′-diyl-bis-(nitrylomethilidyne)]-diphenoxy aluminum isopropoxide (**Ini**) (Scheme 2) on copolymerization of **Lac** with **Tmc**.

We have chosen this initiator because of its bulkiness, hindering chain transfer reactions and cyclization [27]. This feature was recently used by us in preparation of block **Lac**/**Tmc** copolymer by sequential copolymerization using this initiator [28].

The other reason of choosing the indicated initiator is its asymmetry resulting in asymmetry of active chain-ends [29,30,31]. This feature implies possible differences in rates of addition of asymmetric comonomer **Lac** in relation to configuration of active species of growing chain (configuration of residue coming from *R* or *S*-**Ini**). These differences in propagation rate constants result in differences of copolymer structure as shown in the paper.

Due to complexity of the systems discussed in the paper, the reported reactivity ratios are only estimates, depending on the assumed model of copolymerization. They are, however, still useful in predicting of the outcome of **Tmc**/**Lac** copolymerization in dependence on initial conditions.

## 2. Materials and Methods

### 2.1. Materials

*l*,*l*-**Lac** (Boehringer Ingelheim, Ingelheim, Germany, >99%) was crystallized from dry 2-propanol and then purified by sublimation in vacuum (10^−3^ mbar, 90 °C). **Tmc** (Boehringer Ingelheim, Ingelheim Germany, >99%) was crystallized from dry THF/ethyl ether mixture (3/1) and sublimed (10^−3^ mbar, 45 °C). THF solvent was purified, as described previously [11]. Aluminum *tris*-isopropoxide used in trimeric form {Al(O*i*Pr)_3_}_3_ (**A3**) was prepared from the commercial alkoxide (Aldrich, Poznan, Poland, 98%) as described elsewhere [32,33]. Bidentate initiator precursor, asymmetric Schiff’s base derivative, (*R*)-(-) and (*S*)-(+)-2,2′-[1,1′-binaphtyl-2,2′-diyl-bis-(nitrylomethilidyne)]diphenol (**1**), was prepared as described in reference [34].

### 2.2. Polymerization Procedure

All polymerization were performed using the standard high-vacuum technique. The actual initiator, *R*- and/or *S*-**Ini**, was formed in situ using **1** and **A3** in 1.2:1 ratio. The mixture was kept for 24 h in THF as a solvent at 80 °C just before use to ensure complete transformation of precursors to **Ini**.

The 20% excess of **1** did not affect the course of polymerization, both kinetics nor product structure. The mixtures of comonomers and initiator in THF were prepared at room temperature in the special glass vessels in a vacuum. Then the reaction mixture was distributed into several small glass ampoules and/or into a dilatometer, and placed in thermostat at 80 °C. Both homo- and copolymerization experiments have shown that isopropanol present in the initiating mixture acted as effective chain-transfer agent. The observed number average molar masses *M*_n_ were approximately equal as expected for all ^i^PrO- groups initiating chain growth, *M*_n_ = (*M*_Tmc_[**Tmc**]_0_ + *M*_Lac_[**Lac**]_0_)/(3[Al(O*^i^*Pr)_3_]_0_) (*M*_Tmc_ = 102 and *M*_Lac_ = 144 are molar masses of corresponding monomers). On the other hand, the dispersities observed for homopolymers were only slightly higher than expected for processes without side reactions (about 1.1–1.2) and those observed for copolymers were significantly higher (about 1.5–1.6), indicating probably not very fast rate of exchange of chains bearing different terminal units at aluminum active centers (see discussion of results).

Homopolymerizations of **Tmc** and **Lac** were used as reference in kinetic analysis, including molar volume contraction coefficients (CC) for dilatometry. Homopolymerization of monomers were carried out in THF at 80 °C with (*S*)-(−)- and (*R*)-(+)-2,2′-[1,1′-binaphtyl-2,2′-diyl-bis-(nitrylomethilidyne)]diphenoxy aluminum isopropoxide (**Ini**). The starting concentrations of components were as follows:

[**Lac**]_0_ = 1.2 mol·L^−1^, [**Tmc**]_0_ = 2 mol·L^−1^, and [**Ini**]_0_ ≈ 0.002 mol·L^−1^. The conversion of **Lac** in both homo- and copolymerizations was monitored by polarimetry while conversion of **Tmc** in homopolymerizations was monitored with dilatometry and in copolymerizations it was determined at various reaction times from a complex analysis of copolymerization kinetics, consistent with kinetics of changes of dilatometer meniscus level, as described below.

The resulting (co)polymers were isolated by precipitation into cold methanol, and dried in vacuum at room temperature to a constant mass. For comparative studies two poly(trimethylene carbonate) (PTmc) and two poly(l-lactide) (PLac) homopolymers, i.e., PTmcs with *M*_n_ of 33.8 × 10^3^ and 32.1 × 10^3^ (*Ð* ≈ 1.5) as well as PLacs with *M*_n_ equal to 23.5 × 10^3^ and 22.2 × 10^3^ (*Ð* = 1.4 and 1.8 respectively), have been prepared.

**Scheme 3:**
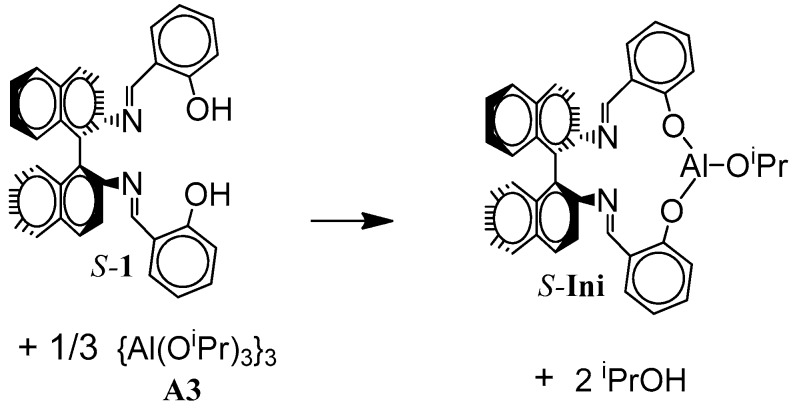
In situ synthesis of *S*-**Ini**. Similarly *R*-**Ini** was formed.

Copolymers of **Lac** and **Tmc** were obtained in ring-opening copolymerization, initiated with (*S*)*-*(−)*-***Ini** or (*R*)*-*(+)*-***Ini**, or with the chosen mixture of both initiator enantiomers, at 80 °C in THF. All experiments were carried out with identical initial concentrations of **Tmc** and initiator: [**Tmc**]_0_ = 2 mol·L^−1^ and [**Ini**]_0_ = 0.002 mol·L^−1^ (prepared in situ, cf. Scheme 3: concentration of growing chains equal to 0.006 mol·L^−1^ due to initial presence of ^i^PrOH, acting as an effective chain transfer agent). Concentration of [**Lac**]_0_ varied from 0.3 to 1.2 mol·L^−1^.

### 2.3. Carbon Nuclear Magnetic Resonance (^13^C NMR)

Composition and microstructure of copolymers were determined by NMR spectroscopy. ^13^C NMR spectra were recorded on a Bruker (Billerica, Massachusetts, USA) AVANCE III (apparatus operating at 500 MHz) in CDCl_3_ (99.8% D) as the solvent. The sample solutions were prepared by dissolving 15–30 mg of dried polymer in 1 mL of CDCl_3_. ^13^C NMR spectra were recorded with inverse gated decoupling, allowing one to minimize the errors of quantitative analyses.

### 2.4. Size Exclusion Chromatography (SEC)

The SEC chromatograph was composed of Agilent (Santa Clara, CA, USA) 1100 isocratic pump, MALLS DAWN EOS photometer (Wyatt Technology Corporation, Santa Barbara, CA, USA) and Optilab Rex differential refractometer (Wyatt Technology Corporation, Santa Barbara, CA, USA). Two PL Gel 5-μm MIXD-C columns were used in a series for separation. Methylene chloride was used as a mobile phase at a flow rate of 0.8 mL·min^−1^. The measurements were conducted at 27 °C. The calibration of the DAWN EOS was performed using p.a. grade toluene, and normalization was performed using a polystyrene standard (PS: *M*_n_ = 3.0 × 10^4^, Polymer Standards Service). The ASTRA 4.90.07 software package (Wyatt Technology Corporation, Santa Barbara, CA, USA) was used for the data collection and processing. *d*n/*d*c increments of the refractive index were determined at λ = 658 nm, as 0.048 and 0.035 mL·g^−1^ for PTmc and PLac, respectively. Samples (100 μL) were injected as solutions in methylene chloride.

### 2.5. Polarimetry

A Perkin-Elmer (Waltham, MA, USA) 241 MC polarimeter was employed for the optical rotation measurements. The optical rotations (OR) of the living polymerization mixtures were measured at 578 nm at room temperature. The instantaneous **Lac** concentrations were determined, assuming additivity of the optical rotations for **Lac** (ORM = 270°) and poly-**Lac** (ORP = 166°), i.e., [LA] = [LA]_0_(OR-ORP)/(ORM-ORP).

### 2.6. Dilatometry

(Co)polymerizations of **Tmc** were carried out in dilatometers equipped with capillary tubes. Dilatometers, of volume about 5 mL, precisely measured, were put in the thermostated water bath in order to perform accurate measurements of the volume changes during polymerization, calculated from measurements of the meniscus level in the capillary tube, diameter 2 mm, with accuracy 0.01 mm. The instantaneous **Tmc** concentrations in polymerization were determined assuming additivity of the polymer and monomer density, using the corresponding equation: [**Tmc**] = [**Tmc**]_0_Δ*H*/Δ*H*_max_, where Δ*H* and Δ*H*_max_ are the changes of meniscus level at the given reaction time and when reaching total conversion of **Tmc**, respectively (small **Tmc** equilibrium concentration was neglected).

The attempts to determine in the same way consumption of **Tmc** in copolymerization failed because the molar volume-contraction-coefficients (CC) for **Tmc** units located in different triads appeared to be not equal. The elaborated method for determining **Tmc** consumption in copolymerization from dilatometric data, based on fitting of simulated copolymerization conversions to experimental ones, is described in details in the Supporting Information section.

### 2.7. Computer Simulations

Kinetics of studied copolymerizations were analyzed comparing experimental data with computer simulations carried out on personal computer with Intel Core i7-975 processor working at frequency 3.33 GHz, 12 GB RAM memory, under Microsoft Windows 7 Pro 64-bit operating system.

Two types of numerical computations were used. Numerical integrations of kinetic differential equations were performed in Matlab v. 7.10 (Natick, MA, USA), adopting the Matlab function ode15s, while parameter fitting performed using the function fminsearch. For more details see the Supporting Information. The computational times of fitting kinetic parameter to experimental data varied between 2 and 48 h, depending on the number of fitted parameters. Monte-Carlo computations were performed using in-house prepared computation programs, according to algorithm devised by Gillespie [35]. Programs were written in Delphi and compiled under Delphi XE2 environment (Embarcadero, Austin, TX, USA). Times of simulations varied in a range of 2–48 h, depending on the number of simulated chains, selected kinetic parameters, and on accounting or neglecting of depropagation reactions.

## 3. Results

In this work, we determined that the relative reactivities of **Tmc** and **Lac** in the copolymerization differ considerably from their reactivities in homopolymerizations, similarly as it was observed for ε-caprolactone/**Lac** systems initiated with the same initiator **Ini** [32]. Significant discrepancies in reactivities of active centers differing in configuration of the used initiator were already observed in **Lac** homopolymerization studies [31]. It stems from different diastereomeric arrangements at the end of growing chains formed by asymmetric **Lac** terminal unit (*S* configuration) and residue from *R*- or *S*-**Ini**. Results of our reference **Lac** homopolymerizations, performed for [**Lac**]_0_ = 1.2 mol·L^−1^, are shown in Supporting Information, confirming large differences in rates of polymerization. On the other hand, one cannot expect any differences in **Tmc** homopolymerization rates, what was confirmed experimentally (initial rate coefficients equal to about 0.088 and 0.093 (±5%) L·mol^−1^·s^−1^, for polymerizations initiated with *R*-**Ini** and *S*-**Ini**, respectively). Therefore, we could expect that the copolymerization reactivity ratios change by altering the active-center initiator-residue configuration, what can result in quite different copolymer structures. In fact, the differences in reactivity ratios were larger than expected by us.

This striking phenomenon is of general importance, since it provides a useful tool for tuning the resultant copolymer microstructure and properties.

### 3.1. Outlook of General Features of Copolymerization Kinetics

**Scheme 4:**
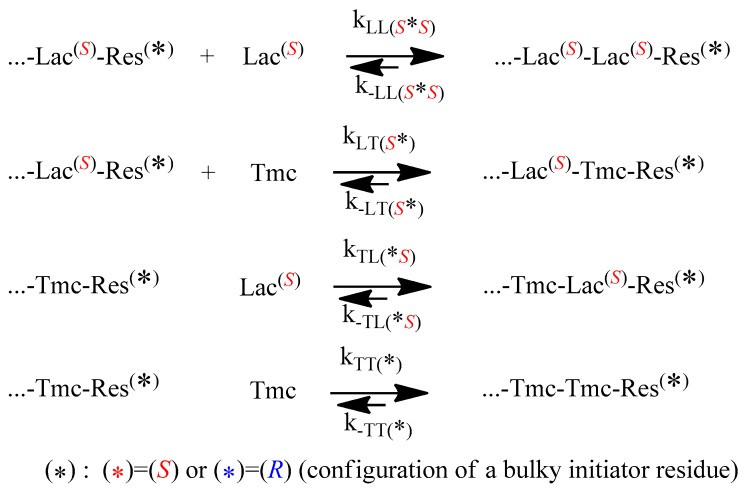
Chemical reactions governing copolymerization of **Lac** with **Tmc**. ‘Res’ denotes the asymmetric residue coming from initiator.

The propagation and depropagation reactions, describing the studied copolymerization, are shown in Scheme 4.

One can note that diastereomeric arrangements imply differences in propagation and depropagation rate constants, in relation to configuration of used initiator, with the exception of the last reversible reaction in the Scheme 4: homopropagation and corresponding depropagation of **Tmc** (apparently no diastereometry, one can expect identical reactivity of enantiomeric **Tmc**-**Res** *).

**Scheme 5:**
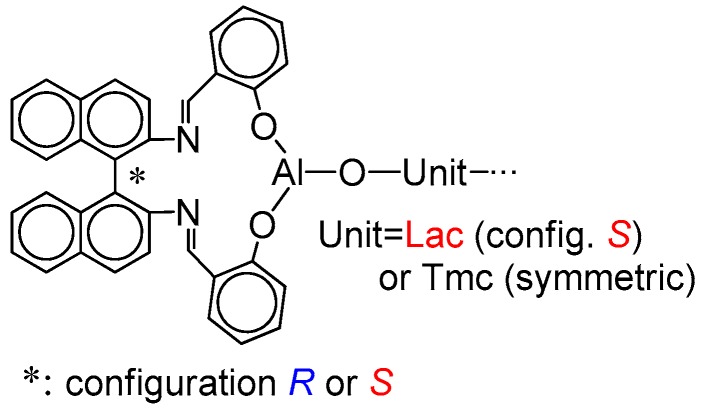
Schematic presentation of the active chain-end in copolymerization of **Lac** with **Tmc**.

The structure of the active species is shown in Scheme 5. If the copolymer unit is symmetric (**Tmc**), one can expect that the rate of insertion of alike monomer molecule into Al-O-Unit bond does not depend on configuration of initiator residue **Res** at the chain-end. On the other hand, addition of asymmetric monomer molecule (**Lac**) depends on configuration of **Res** because we can have two different diastereomeric arrangements here. Similarly, when Unit is asymmetric (**Lac**), addition of any of comonomers (**Lac** or **Tmc**) depends on configuration of **Res** (reactions involving diastereomeric active species).

Thus, depending on the configuration of **Ini**, copolymerization can proceed in a different way.

In principle, copolymerization kinetics can be monitored by any method giving access to comonomer conversions. Unfortunately, spectroscopic methods we considered (UV, IR, ^1^H NMR) could not be used effectively because of the lack of sufficiently separated signals of the comonomers and the copolymer. Only ^13^C NMR spectra could give the corresponding information. Unfortunately, due to technical problems (taking samples from the reaction mixture avoiding its contamination, followed by isolation of product) only a few kinetic data points could be obtained. Much more convenient methods seemed polarimetry for following conversion of **Lac** and dilatometry for following conversion of **Tmc** (polymerization of **Lac** results in no change of the reaction system volume), following the approach, applied by Florczak and Duda in analysis of copolymerization kinetics of **Lac** with ε-caprolactone [32].

However, it appeared that following conversion of **Tmc** with dilatometry was not straightforward. We have observed that changes of the system volume were significantly larger than expected on the basis of contraction coefficients determined from homopolymerization of **Tmc**. Moreover, conversion of **Tmc** determined from dilatometry in a standard way was significantly different from that obtained from ^13^C NMR, available for a few reaction times of one copolymerization system, initiated with the mixture of *R*- and *S*-**Ini** (cf. below in the corresponding section).

Consequently, we came to conclusion that contraction coefficients for **Tmc** and, possibly, also for **Lac** units, depend on the type of triad in which the given unit occupies the central place. Thus, in order to be able to use dilatometry for following the **Tmc** conversion, we had to determine, or at least estimate, three values of contraction coefficients for any of comonomers, e.g., for A unit coefficients for homotriad AAA, heterotriad BAB, and the average value for asymmetric triads AAB and BAA. The average value for asymmetric triads is sufficient because in copolymer of sufficiently long chains the numbers of triads AAB and BAA are virtually equal.

However, in order to estimate these parameters directly we would have to have a sufficiently large number of experimental data describing the relationship between comonomer conversions and copolymer microstructure (triad level), and volume contraction corresponding to the given samples.

Unfortunately, ^13^C NMR did not give the sufficient information about triad (nor dyad) contributions, because some triads, assigned according to Dobrzynski and Kasperczyk [23], overlap (e.g., of triads Tmc**LL**Tmc, LacL**L**Tmc, and Tmc**L**LLac: LL means here **Lac** unit, composed of two lactic units L, in bold are marked the lactic units relevant to overlapping signals, cf. Table 1).

Signals of copolymer structures stemming from segmental exchange (e.g., of the isolated lactic unit Tmc**L**Tmc) were observed by us only in spectra of systems kept more than 24 h after completing copolymerization which confirmed our assumption that reshuffling can be neglected.

**Scheme 6:**
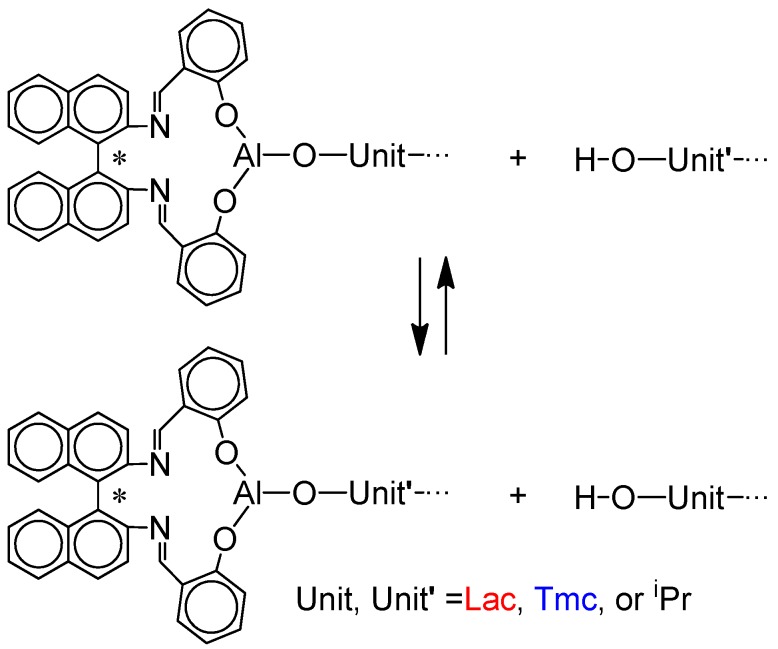
Chain-transfer reactions operating in the studied copolymerization systems. Due to the assumption of instantaneous initiation isopropyl (^i^Pr) containing active species, as well as ^i^PrOH, coming from the in situ synthesis of **Ini** (cf. Scheme 3), could be neglected.

We managed to estimate the contraction coefficients from analysis of copolymerization kinetics. Unfortunately, some assumptions, leading eventually to estimates valid only for the assumed model of copolymerization, had to be adopted. These assumptions were as follows:

1. Kinetics of copolymerization follows the reactions as reported in Scheme 4 and depolymerizations can be neglected up to at least 80% of conversion. The last is based on our simulations of reversible copolymerizations [36], indicating that depolymerizations with low equilibrium concentrations of comonomers are usually negligible in most systems up to conversions about 90%.
2. The chain-transfer reactions involving hydroxyl containing chains are fast, allowing one to neglect them in kinetic analysis and consider all chains terminated with the given unit kinetically indistinguishable. Alcohol chain end-groups in the copolymerization systems are formed at the very beginning of copolymerization due to the fact that we initiated our systems with the in situ formed **Ini** (Scheme 3), what resulted in formation of isopropanol, which also can initiate copolymer chains via chain-transfer processes (Scheme 6). The chain-transfer reactions were, however, taken into account in more detailed kinetic analysis, allowing to get better agreement of experimental and simulated dispersities of copolymers.
3. Instantaneous initiation gives living unimers prior to any propagation reactions. This approximating assumption allows to neglect initiation reaction in kinetic analysis.

These assumptions allowed us to describe copolymerization systems entirely by the kinetic Scheme 4, not accounting OH-terminated chains. Such a simple model of copolymerization appeared to be useful if copolymerization main features, such as copolymer composition and microstructure, are concerned. However, when more detailed features, like molar mass distribution are to be analyzed, the copolymerization model including the rate of exchange of OH-terminated chains with ones bearing active species, has to be used, as shown below in the paper.

Nevertheless, kinetics of irreversible copolymerizations following Scheme 4 (with all depropagation rate constant equal to zero) can be predicted from integration of the corresponding kinetic differential equations. On the other hand, Monte Carlo simulations, taking into account depropagations, could confirm validity of neglecting them, as well as could give access to detailed description of copolymer microstructure.

**Figure 1:**
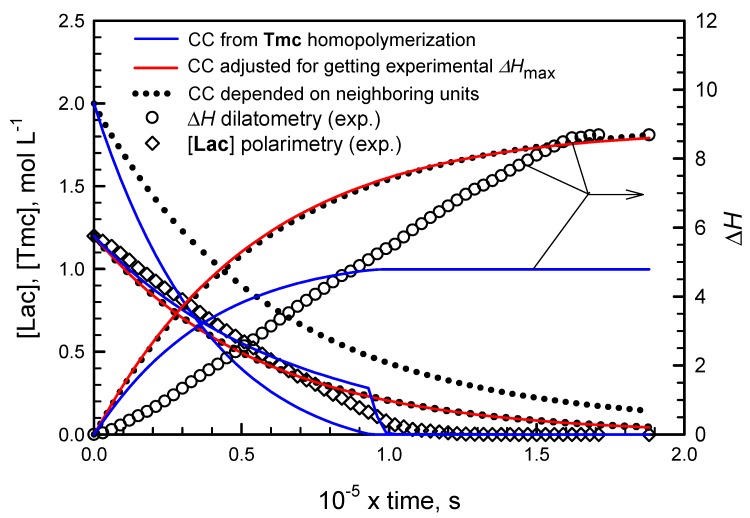
Fitting of simulated changes of [**Lac**] (lines starting from [**Lac**] = 1.2) and of the dilatometer meniscus height Δ*H* (lines starting from Δ*H* = 0) to experimental data (diamonds: [**Lac**], circles: meniscus Δ*H*) for copolymerization of **Lac** with **Tmc**, initiated with [*S*-**Ini**] = 2 × 10^−3^ (+[^i^PrOH]_0_ = 4 × 10^−3^ mol·L^−1^ due to in situ synthesis of **Ini**), [**Lac**]_0_ = 1.2, [**Tmc**]_0_ = 2 mol·L^−1^. Blue lines: volume contraction coefficient (CC) for **Tmc** copolymerization equal to that determined from homopolymerization experiments, red lines: CC for **Tmc** units increased to get the same final meniscus height as determined experimentally, black dot lines: **Tmc** and **Lac** unit CC depending on neighboring units, as determined in our studies (see Supporting Information). Additionally the predicted changes of [**Tmc**] are presented (lines starting from [**Tmc**] = 2).

Applying numerical integration of differential kinetic equations we could predict evolution of copolymerization in time. However, attempts to fit rate coefficients failed, indicating that comonomer consumption rates seemed not to decrease with comonomer concentrations as expected. Rates of consumption of comonomers were initially lower than those predicted from simulations and eventually higher: the apparent rate coefficients seemed to increase with conversion. An example of such fitting is given in Figure 1. More details concerning this type of kinetic analysis are given in Supporting Information.

The presented plots suggest that apparent rate coefficients change with conversion, being initially lower than obtained from parameter fitting (experimental slope for evolution of [**Lac**] initially lower than obtained in simulations) and, at the end of comonomer consumption, the rate coefficients seem to be higher than obtained from simulations (the corresponding slope for experimental points for reaction times longer than 1000 min is higher than that of fitted plots).

Important is also observation that volume contraction coefficients for copolymer units differ from those observed in homopolymerizations. Either contraction coefficients for **Tmc** unit depend on other units neighboring the given one, or the same can be said about **Lac** units (contraction coefficients for some triads with **Lac** located in the middle not equal to zero as in homopolymerization), or contraction coefficients for both types of copolymer units depend on copolymer microstructure. Our fitting analysis (see below and Supporting Information) suggest that the third possibility is true.

Analyzing simulated kinetic curves in comparison to experimental ones we deduced that the reaction medium, continuously changing while concentrations of comonomers and copolymer units evolve, makes the rate coefficients depend on conversion. It can be understood as both comonomer molecules and copolymer units solvate active species in varying proportions at different conversions. As **Lac** molecule and copolymer units are asymmetric one cannot exclude some diastereomeric effect making the apparent rate coefficients dependent on conversion.

In order to get a relatively simple kinetic model of copolymerization consistent with experimental data we have assumed that the relative changes of apparent rate coefficients with conversion are approximately identical for all reactions, changing simultaneously, resulting in constant ratios of rate constants. The analyzed models of copolymerization and methods of fitting apparent relative rate coefficients to experimental data are described in detail in Supporting Information. Here we only indicate the main result of this analysis. Namely, if the abovementioned assumption is valid then the kinetics presented in conversion scale is characterized by kinetic parameters independent of conversion or reaction time. These kinetic parameters are ratios of instantaneous (changing) rate coefficients of reactions operating in the system. One can choose different ratios to describe the analyzed copolymerizations but our choice was given as below.

Initially, we related all rate constants to the **Tmc** homopropagation rate constant *k*_TT_ (see Scheme 4), chosen as the rate constant presumably independent of configuration of **Ini** and denoted the corresponding ratios *z*_XY_ = *k*_XY_/*k*_TT_, where X, Y is L and/or T, which stand for **Lac** or **Tmc** comonomer/unit.

However, one can easily find that one can relate these *z*_XY_ parameters (XY different than LL) with *z*_LL_ and standard parameters such as reactivity ratios and, for depropagation rate constants, additionally with the equilibrium constants. These relationships are presented in the equation set (1). Starting from this equation set we use letters L and T, while denoting with **Lac** and **Tmc** comonomers/comonomer units, respectively. Besides, in all equation sets we use for these letters red and blue color, respectively. The same colors are used in some Figures and plots describing copolymer units or blocks related to the studied comonomers.

(1) $$\begin{array}{l} z_{\mathrm{LL}}=\frac{k_{\mathrm{LL}}}{k_{\mathrm{TT}}}\;,\;z_{\mathrm{TT}}=\frac{k_{\mathrm{TT}}}{k_{\mathrm{TT}}}=1\\ z_{LT}=\frac{k_{LT}}{k_{\mathrm{TT}}}=\frac{k_{LT}}{k_{\mathrm{LL}}}\times \frac{k_{\mathrm{LL}}}{k_{\mathrm{TT}}}=\frac{z_{\mathrm{LL}}}{r_{L}}\;,\;z_{TL}=\frac{k_{TL}}{k_{\mathrm{TT}}}=\frac{1}{r_{T}}\\ z_{-\mathrm{LL}}=\frac{k_{-\mathrm{LL}}}{k_{\mathrm{TT}}}=\frac{k_{-\mathrm{LL}}}{k_{\mathrm{LL}}}\times \frac{k_{\mathrm{LL}}}{k_{\mathrm{TT}}}=\frac{z_{\mathrm{LL}}}{K_{\mathrm{LL}}}\;,\;z_{-\mathrm{TT}}=\frac{k_{-\mathrm{TT}}}{k_{\mathrm{TT}}}=\frac{1}{K_{\mathrm{TT}}}\\ z_{-LT}=\frac{k_{-LT}}{k_{\mathrm{TT}}}=\frac{k_{-LT}}{k_{LT}}\times \frac{k_{LT}}{k_{\mathrm{LL}}}\times \frac{k_{\mathrm{LL}}}{k_{\mathrm{TT}}}=\frac{z_{\mathrm{LL}}}{K_{LT}r_{L}}\;,\;z_{-TL}=\frac{k_{-TL}}{k_{\mathrm{TT}}}=\frac{1}{K_{TL}r_{T}} \end{array}$$

Thus, we could formulate the kinetic differential equations in conversion scale, with only the limited number of the mentioned relative kinetic parameters: *z*_LL_ and reactivity ratios *r*_L_ and *r*_T_ (and for not negligible depropagations also the equilibrium constants), taking into account the following relationships (formulated here for irreversible copolymerization):

(2) $$\begin{array}{l} Conv=\int_{0}^{t}\frac{\left(-\frac{d[L]}{dt}-\frac{d[T]}{dt}\right)}{{\left[L\right]}_{0}+{\left[T\right]}_{0}}dt\\ \frac{dConv}{dt}=\frac{\left(-\frac{d[L]}{dt}-\frac{d[T]}{dt}\right)}{{\left[L\right]}_{0}+{\left[T\right]}_{0}}=\frac{k_{\mathrm{LL}}[L*][L]+k_{TL}[T*][L]+k_{\mathrm{TT}}[T*][T]+k_{LT}[L*][T]}{{\left[L\right]}_{0}+{\left[T\right]}_{0}}\\ \frac{d[\;]}{dConv}=\frac{\frac{d[\;]}{dt}}{\frac{dConv}{dt}}=f\left([L],\;[T],\;\ldots {\;,\;z}_{\mathrm{LL}}{,\;r}_{L}{,\;r}_{T}\right) \end{array}$$

where [ ] in Equation (2) corresponds to concentration of any reagent, species, or copolymer sequence in copolymer. For instance, the corresponding equations formulated for evolution of concentrations of **Lac** (L) monomer and LT dyads are given by equation set (3):

(3) $$\begin{array}{l} \frac{d[L]}{dConv}=-\left({\left[L\right]}_{0}+{\left[T\right]}_{0}\right)\times \frac{z_{\mathrm{LL}}[L*][L]+{[T*][L]/r}_{T}}{z_{\mathrm{LL}}[L*][L]+{[T*][L]/r}_{T}+[T*][T]+z_{\mathrm{LL}}{[L*][T]/r}_{L}}\\ \frac{d[LT]}{dConv}=\left({\left[L\right]}_{0}+{\left[T\right]}_{0}\right)\times \frac{z_{\mathrm{LL}}{[L*][T]/r}_{L}}{z_{\mathrm{LL}}[L*][L]+{[T*][L]/r}_{T}+[T*][T]+z_{\mathrm{LL}}{[L*][T]/r}_{L}} \end{array}$$

Applying the formulated set of kinetic differential equations in conversion scale (see Supporting Information) to experimental data allowed us to predict main features of copolymers obtained in the studied systems.

### 3.2. Dependence of Copolymer Structure on Configuration of **Ini**

**Figure 2:**
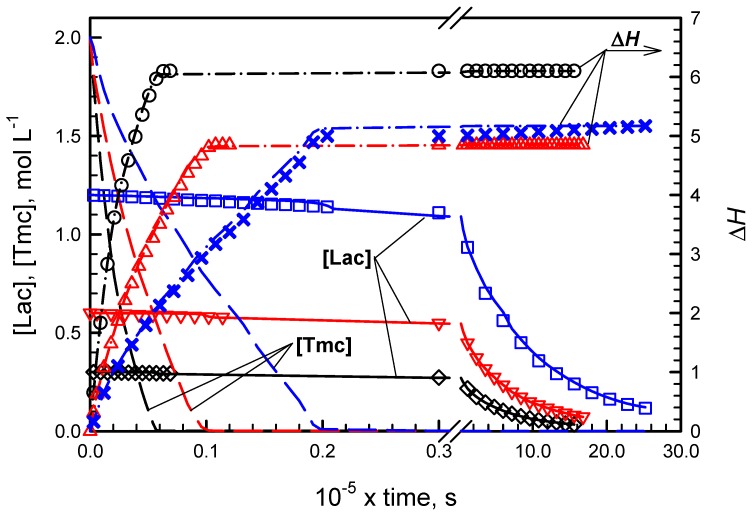
Experimental and simulated evolution of [**Lac**], [**Tmc**], and Δ*H* in copolymerizations initiated with *R*-**Ini** for different [**Lac**]_0_: 0.3 (black), 0.6 (red), and 1.2 mol·L^−1^ (blue). Experimental data marked by symbols, simulated data by lines. Other initial conditions: [**Tmc**]_0_ = 2 mol·L^−1^. [*R*-**Ini**]_0_ = 2 × 10^−3^ mol·L^−1^ (+ [^i^PrOH]_0_ = 4 × 10^−3^ mol·L^−1^ due to in situ synthesis of **Ini**).

**Figure 3:**
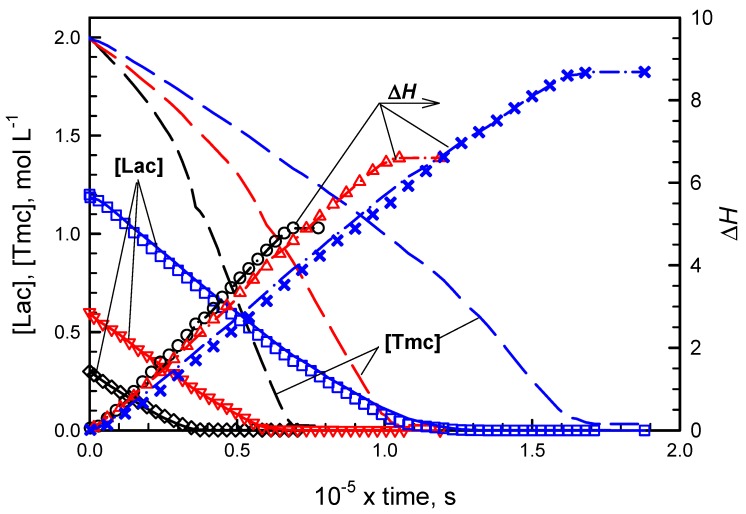
Experimental and simulated evolution of [**Lac**], [**Tmc**], and Δ*H* in copolymerizations initiated with *S*-**Ini**. Plots description and copolymerization conditions as given in caption for Figure 2, but *R*-**Ini** enantiomer was used.

Figure 2 and Figure 3 present conversions of comonomers in copolymerizations initiated with *R*- and *S*-**Ini**, respectively. Conversions of **Lac** were detected directly due to polarimetric measurements while conversions of **Tmc** were obtained from the elaborated kinetic analysis presented in Supporting Information. The kinetic and contraction parameters were fitted to describe simultaneously three copolymerization experiments initiated with the given enantiomer of **Ini**, differing with the initial **Lac** concentrations.

While in systems with *R* initiator enantiomer, initially mostly **Tmc** is consumed, giving presumably the product resembling diblock copolymer, in systems with *S*-**Ini** we obtained product containing in the initial parts of chains both comonomers in similar quantities. The chains are terminated eventually with **Tmc** blocks due to the fact that **Lac** was consumed before **Tmc**, used in significant excess. Kinetic analysis confirms this description of products giving both reactivity ratios much higher than unity in *R*-**Ini** systems and the product of reactivity ratios lower than unity in *S*-**Ini** systems.

**Figure 4:**
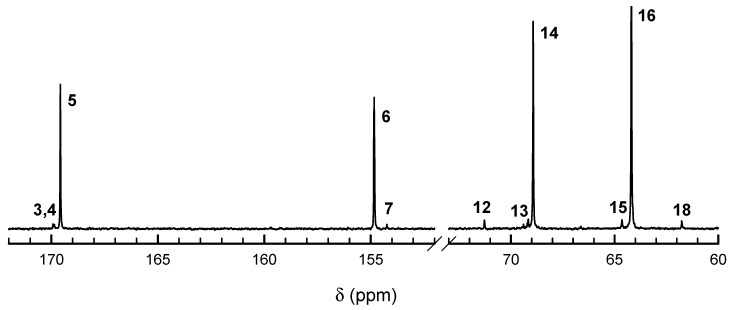
^13^C NMR spectra of **Tmc**/**Lac** copolymers prepared with *R*-**Ini**. Polymerization conditions: [**Lac**]_0_, [**Tmc**]_0_, [*R*-**Ini**]_0_ = 1.2, 2, and 2 × 10^−3^ (+[^i^PrOH]_0_ = 4 × 10^−3^) mol·L^−1^, respectively. Time 3.81 × 10^6^ s, conversion **Lac** = 92%, conversion **Tmc** = 99%. Note large contributions of signals of homo **Lac** and **Tmc** sequences, signals 13, and 15, respectively. Assignment of signals is given in Table 1.

**Figure 5:**
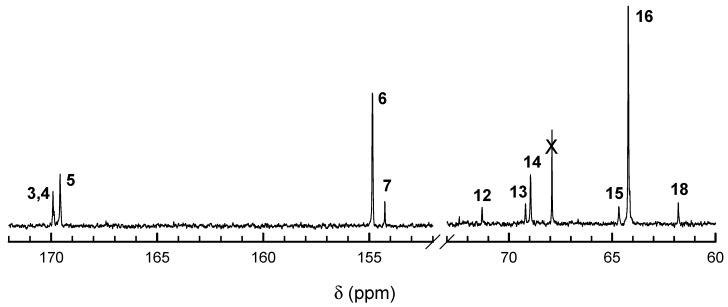
^13^C NMR spectra of **Tmc**/**Lac** copolymers prepared with *S*-**Ini**. Polymerization conditions: [**Lac**]_0_, [**Tmc**]_0_, [*S*-**Ini**]_0_ = 1.2, 2, and 2 × 10^−3^ (+[^i^PrOH]_0_ = 4 × 10^−3^) mol·L^−1^, respectively. Time 1.49 × 10^5^ s, conversion **Lac** = 96%, conversion **Tmc** = 99%. Note a relatively low contribution of a signal of homo **Lac** sequences (signal 13), and quite large contribution of TmcLacTmc sequence (signal 10). Assignment of signals is given in Table 1.

^13^C NMR spectra (Figure 4 and Figure 5) also confirm the above description of copolymers, indicating the large excess of homodyads in products initiated with *R*-**Ini** and a low quantity of homodyads LacLac in copolymer initiated with *S*-**Ini**, what indicates some tendency to alternacy, as expected from the estimated product of reactivity ratios being significantly lower than unity. Assignment of ^13^C NMR signals is given in Table 1.

Our kinetic analysis allowing us to estimate the reactivity ratios for the studied systems is described in details in Supporting Information. It was based on fitting of the simulated evolution of the studied systems, described by the formulated differential kinetic equations with the relative kinetic parameters, including reactivity ratios, to experimental evolution of the studied systems.

Main results of the primary kinetic analysis are given in Table 2 while the Figure 2 and Figure 3 present experimental and computed from kinetic analysis evolution of copolymerizations initiated with *R* and *S* enantiomers of **Ini**.

Rather unexpected was the finding that the difference between **Tmc** reactivity ratios in relation to configuration of **Ini** (*r*_T(*R*)_ > *r*_T(*S*)_) is as large as three orders of magnitude. It stems probably from strong steric hindrance of *R*-**Ini** residue of active species while the transition state for **Lac** addition to **Tmc** * is formed. The corresponding hindrance for **Tmc** addition is smaller because of the lack of methyl groups, present in **Lac**. On the other hand, the smaller difference observed for **Lac** reactivity ratios (*r*_L(*R*)_ > *r*_L(*S*)_) and indicating that addition of **Lac** to **Lac** * is faster than addition of **Tmc** for both configurations of **Ini**, is probably due to relatively large differences in activation enthalpies of the corresponding reactions.

It is important to indicate here that our fitting computations could not estimate the *z*_LL_ parameter in copolymerizations initiated with *S*-**Ini**, nor initiated with the mixture of **Ini** enantiomers. It is so, because of the systems quickly attaining steady state conditions maintaining the proportion of active species. These steady state conditions, applied also while deriving Mayo-Lewis equations, are generally accepted while analyzing copolymerization kinetics. However, when at least one of the reactivity ratios is very high, attaining of the steady state conditions can require quite large conversions. For such systems, here observed for *R*-**Ini** initiated **Lac**/**Tmc** copolymerization, the Mayo-Lewis equations can be regarded as giving only crude estimates for relative rates of comonomer consumptions. In fact, the *z*_LL_ parameter in ‘normal’ copolymerizations determines the steady state condition ratio of concentrations of **Lac** * and **Tmc** * active species, not influencing the relative rates of comonomer consumption. The estimation of the *z*_LL_ = *k*_LL_/*k*_TT_ parameter can be done only applying some specific methods, for instance analyzing in details the molar mass distribution in relation to chain compositions [37].

Thus, in our simulations, when fitting the relative kinetic parameters for *S*-**Ini** systems by minimization of the defined objective function (see Supporting Information), we observed, as expected, independence of the fitting results for *r*_L_ and *r*_T_, for any assumed value for *z*_LL_ in the range between 10^−2^ and 10^2^ (not checked outside this range). 

On the other hand, due to non-steady state conditions up to high conversions, while fitting relative parameters for *R*-**Ini** systems we could attain minima of the objective function (see Supporting Information), giving different results for *r*_L_ and *r*_T_, for any assumed value of *z*_LL_. Besides, also due to slowly attaining the steady state conditions, the fitting results depended on the assumed proportion of initiating unimers. The observed minima were on quite similar levels for *z*_LL_ in the range between 10^−3^ and 0.5, being on apparently higher levels outside this range. Thus, our estimates of the reactivity ratios *r*_L_ and *r*_T_ and of the relative rates of comonomer consumption for *R*-**Ini** systems, given in the Table 2, are rather crude. They were obtained for *z*_LL_ equal to 4 × 10^−3^, giving only slightly lower the objective function minimum than observed for different *z*_LL_ in the indicated range. Moreover, these estimates were obtained assuming initiation exclusively with **Tmc** unimers. The last assumption was made because of the observed large differences of rates of homopolymerization of **Tmc** and **Lac** initiated with *R*-**Ini**, **Tmc** polymerizing much faster, see Supporting Information.

The results presented in Table 2 indicate tremendous differences between copolymerization systems differing only in configuration of asymmetric bulky initiator. While *R*-**Ini** gives copolymer of virtually oligoblock structure: initially mostly only **Tmc** is consumed, forming the corresponding blocks, and next the blocks of poly-**Lac** are formed. The product of reactivity ratios is very high, indicating that practically negligible inserts of **Lac** units in the initial, mostly homo-**Tmc** parts of chains, are short blocks of **Lac** rather than separate single **Lac** units. Similarly, at the end parts of chains, being approximately the **Lac** homo-blocks, one can expect only infrequent inserts of short homo-blocks of **Tmc**. However, due to a very high *r*_L_, similarly as of *r*_T_, and *k*_LL_ << *k*_TT_, (*z*_LL_ estimated to be about 10^−2^) some homo-**Lac** chains, if formed in the initial part of copolymerization, grow slower than chains formed from **Tmc** unimers. Some of these chains can survive till the end of copolymerizations, forming small, not negligible for systems with not sufficiently high *DP*_n_, fractions of homo **Lac** polymer, differing in the average molar mass from copolymer chains. One cannot also exclude formation of a fraction of homo-**Tmc** polymer in some copolymerization systems. This characteristics of *R*-**Ini** copolymerization systems results in dispersity of product significantly higher than expected for random copolymerizations proceeding without side reactions like, for instance, segmental exchange or cyclizations. Even if one assumed that the system is initiated only with **Tmc** unimers (as done by us in simulations giving *r*_L_ and *r*_T_ values for *R*-**Ini** systems in Table 1), dispersity can be quite high, due to slow transformation of **Tmc** * chains into **Lac** * chains. This phenomenon resembles slow initiation, which also leads to broadened dispersity.

On the other hand, using as initiator the *S*-**Ini** results in initial rates of consumption of both comonomers not differing much and in some tendency to alternacy (product of reactivity ratios about 0.1). Due to the excess of **Tmc** in all studied copolymerization systems one can expect copolymer chains ended with homoblocks of **Tmc** containing some small amount of inserts of separated units of **Lac**.

Our Monte Carlo simulations confirm the above description of copolymer chains, deduced from the estimated reactivity ratios for products obtained in the studied systems. 

However, there was one experimental inconsistency in numerical simulations with experimental data. Namely, dispersity of copolymer chains initiated with *S*-**Ini** (fast inter-transformations of **Lac** * and **Tmc** * active species), are significantly higher than expected from simulations assuming, as mentioned above, fast exchange of active chain-ends with hydroxyl terminated ones. A relatively large dispersity (above 1.5 for higher conversions) was successfully explained by the rate of the exchange reactions involving OH terminated chains (Scheme 6) being not sufficiently high. Therefore, the copolymerization systems do not behave exactly, as expected from simulations assuming the discussed until now model. Verifying this hypothesis with Monte Carlo simulations, we came to the conclusion that the hydroxyl terminated chains are probably transformed into living chains with rates much lower than propagation, resulting in broadening of the molar mass distribution. Numerical simulations, described in Supporting Information, taking into account the slow chain-transfer processes, allowed us to estimate the average relative rate constants of chain-exchange in analyzed copolymerizations *k*_ex_/*k*_TT_ as well as the effective ratio of homopropagation rate constants *k*_LLR_/*k*_LLS_, important for systems initiated with the mixture of **Ini** enantiomers, as described below.

Consequently, the presented below results take into account the chain-end exchange reactions in all studied systems.

**Figure 6:**
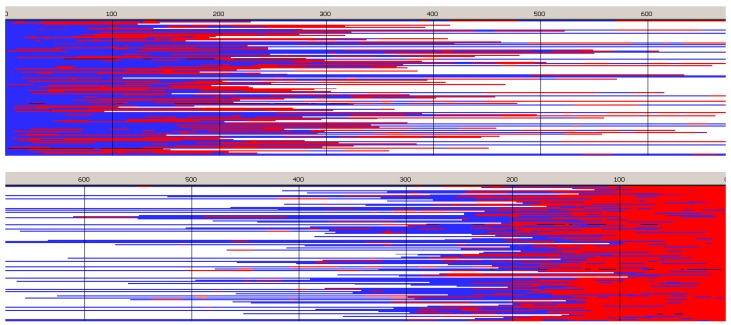
Sample of simulated chains in copolymerization initiated with **Tmc***-R*-**Ini** *. Reactivity ratios as given in Table 2, z_LL_ = 3.8 × 10^−3^, *k*_ex_/*k*_TT_ = 4 × 10^−2^. [**Tmc**]_0_ = 2, [**Lac**]_0_ = 1.2 mol·L^−1^, and [**Ini**]_0_ = 2 × 10^−3^ mol·L^−1^ (+ [^i^PrOH]_0_ = 4 × 10^−3^ mol·L^−1^ due to in situ synthesis of **Ini**). Conversion 95%, *DP*_n_ = 507.4, *Ð* = 1.48, average number of homoblocks per chain equal to 5.2. Red and blue mark **Lac** and **Tmc** units, respectively. Top box: unit positions numerated starting from chain beginning; bottom box: starting from chain-end. For the sake of plots clarity the chains longer than *DP* = 1.3 *DP*_n_ are shown only partially (this applies also to similar plots below).

**Figure 7:**
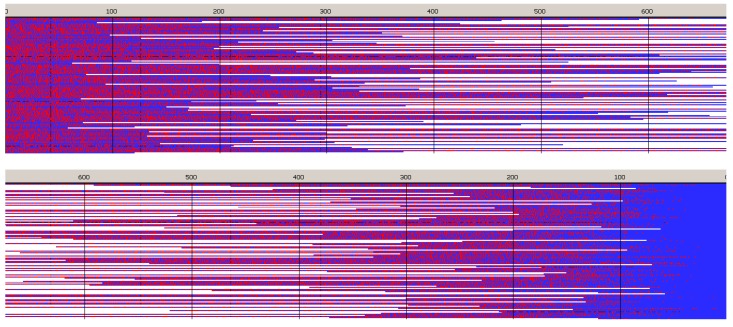
Sample of simulated chains in copolymerization initiated with *S*-**Ini** (mixture of **Lac** * and **Tmc** * unimers). Reactivity ratios *z*_LL_ = 37.6, *k*_ex_/*k*_TT_ = 8.1. Other conditions as in Figure 6. Conversion 95%, *DP*_n_ = 506.4, *Ð* = 1.40, average number of homoblocks in a chain equal to 292.

**Figure 8:**
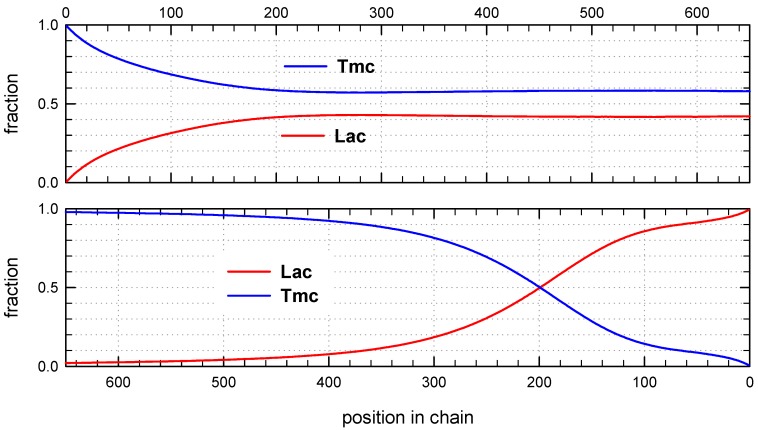
Distribution of copolymer units along an average chain expressed as mole fractions for system initiated with Tmc-*R*-Ini * unimer. The relative kinetic parameters as shown in Table 2 and caption to Figure 6. Unit positions numerated from chain beginning (**top box**) and from the chain-end (**bottom box**).

**Figure 9:**
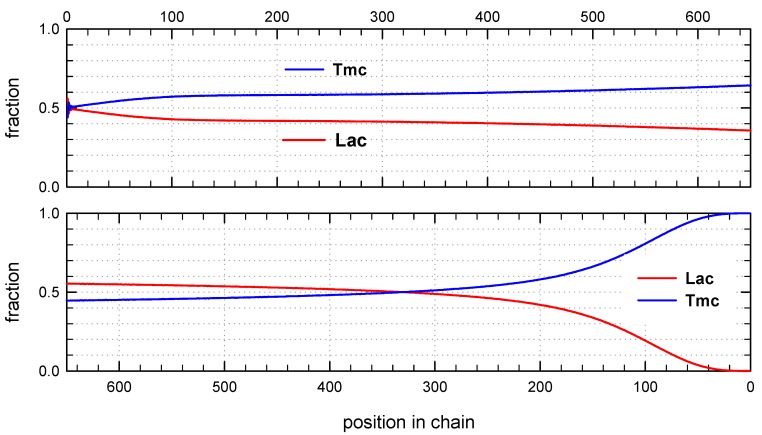
Distribution of copolymer units along an average chain expressed as mole fractions for system initiated with the mixture of **Lac-***S***-Ini** * and **Tmc-***S***-Ini** * unimers. The relative kinetic parameters as shown in Table 2 and caption to Figure 7. Unit positions numerated from chain beginning (**top box**) and from the chain-end (**bottom box**).

Figure 6, Figure 7, Figure 8 and Figure 9 present structures of copolymer chains formed in systems initiated by *S-* or *R-* enantiomer of **Ini**, simulated by MC method neglecting depropagation reactions. Initial concentrations of reagents were the same: [**Tmc**]_0_ = 2, [**Lac**]_0_ = 1.2 mol·L^−1^, and [**Ini**]_0_ = 2 × 10^−3^ mol·L^−1^ (+[^i^PrOH]_0_ = 4 × 10^−3^ mol·L^−1^ due to in situ synthesis of **Ini**). The only difference consisted in initiation by **Tmc** * unimer in case of *R-* enantiomer and mixture of **Tmc** * and **Lac** * unimers (proportionally to comonomer concentrations) in case of *S-*enantiomer. Reactivity ratios used in each simulation are given in Table 2. Top boxes: plots prepared using unit positions numerated starting from chain beginning; bottom boxes: starting units numeration from chain-end.

Copolymer formed with *R*-**Ini** and initiated with **Tmc** * unimers (Figure 6) is a block copolymer, containing on the average 5.2 blocks in a chain. As it was initiated with **Tmc** unimers chains start with **Tmc** blocks and, due to faster consumption of **Tmc**, chains are terminated with **Lac** blocks. 

Monte Carlo simulations allow to present also the average composition of copolymers along average chain (computed for the whole set of chains) as well as similar distribution of homo and hetero dyads. The corresponding plots are shown in Figure 8 and Figure 9.

The corresponding plots differ, being dependent on numeration of unit positions, starting either from the chain beginning or chain-end. Due to rather broad molar mass distribution these differences are quite large. If position of unit is counted from chain beginning the fractions of copolymer units at more distant positions come to plateau and practically does not change up to chain positions at least about twice *DP*_n_. On the other hand, if unit positions are counted from chain-ends, one can clearly see a gradient-like feature of copolymer chains.

Distribution of units in copolymer formed with *S*-**Ini** and initiated with the mixture of **Lac-***S***-Ini** * and **Tmc-***S***-Ini** * unimers, in proportion corresponding to initial concentration of comonomers (1.2:2) (Figure 7) differs significantly from system initiated by **Tmc**-*R*-Ini * unimers.

Initial parts of chains, exceeding up to about 70% of chain length, contain statistical distribution of comonomers, apparently with close to each other proportion of comonomer units, no gradient of comonomer composition along individual chains in these regions were visible. However, when we analyze positions of units and sequences in the whole set of copolymer chains, the contributions of **Lac** and **Tmc** units (Figure 9) change slightly steadily with unit position, indicating gradient feature. The shapes of the corresponding plots differ, depending on that if unit positions are numerated starting from the chain beginning or chain-end, similarly as it was observed for *R*-**Ini** system.

In the Supporting Information one can find the similar plots presenting distribution of dyads and the average lengths of homoblocks along chain for systems initiated with both **Ini** enantiomers. 

The presented copolymer structures Figure 6 and Figure 7 were prepared choosing the exchange relative parameter *z*_ex_ (listed in the Figure captions) to get dispersity close to the observed in experiments. One can observe that *z*_ex_ for *R*- and *S*-**Ini** systems differ significantly. It stems probably not only from different reactivities of **Lac**-*R*-Res* and **Lac**-*S*-Res* species (diastereomers) but also from the simplifying assumption (see Supporting Information) that all relative kinetic exchange parameters are equal, independently on copolymer units neighboring OH group or *R*/*S*-**Ini** residue of active centers.

The rate of chain-end exchange is more important in copolymerization system initiated with the mixture of *R* and *S* enantiomers of **Ini**. It is so because beyond determining copolymer dispersity it predetermines also the effective rate of exchange of the **Ini** residues of different configuration at active chain-ends and consequently the chain reactivity ratios, establishing copolymer microstructure. Any growing chain can, if the exchange is sufficiently fast, change configuration of its active species, with frequency dependent on the discussed relative rate coefficient *z*_ex_.

### 3.3. Copolymerization Initiated with the Mixture of Enantiomers of **Ini**

The observed differences in features of copolymerization systems initiated with *R*-**Ini** and *S*-**Ini** suggests that one can control, to some extent, copolymerization features by using instead of one enantiomer of the studied asymmetric bulky initiator **Ini** the mixture of its enantiomers in variable proportions. 

We performed such an experiment choosing the proportion of initiators *R*-**Ini**:*S*-**Ini** equal to 94:6. The large excess of *R* enantiomer was adopted because the rate of copolymerization initiated with *R*-**Ini**, leading to long homo-blocks, is much lower than that initiated with *S*-**Ini** and, on the other hand, the latter leads to statistical, almost alternating structures. Thus, the *S* enantiomer, although being in minority but with relatively fast cross-propagation rate constants, can effectively change the type of active species from **Lac** * to **Tmc** * and vice versa. The expected result was to obtain relatively homogeneous multi-block chains.

Copolymerization results were only partly as expected. Although the copolymerization rate and proportion of hetero-dyads were higher than in *R*-**Ini** copolymerization (for ^13^C NMR see the Supporting Information) and the average number of copolymer blocks increased, a gradient-like feature is still visible in Monte Carlo simulations. It was explained by rather slow, not sufficiently fast, as we expected, exchange of **Lac**-*R*-**Res** * active species (characterized by high reactivity ratio) into **Lac**-*S*-**Res** * active species (via **Lac**-OH terminating chains, acting as intermediates). If this process was fast enough, **Lac**-*S*-**Res** * could fast attach **Tmc** comonomer (low reactivity ratio), forming **Tmc**-*S*-Res* terminated chain, which can readily attach **Lac**. Similarly, **Tmc**-*R*-**Res** * active species (high reactivity ratio, slow addition of **Lac** comonomer unit), apparently not as fast as we expected, can be transformed (also via OH terminated intermediate) into **Tmc**-*S*-**Res** * attaching next relatively quickly **Lac**, forming **Lac**-*S*-**Res** * species, already discussed. Consequently, contribution of heterodyads, as observed in ^13^C NMR spectrum (Supporting Information), is higher than in copolymer formed with *R*-**Ini**, but instead of approximately homogeneous unit distribution, one can rather expect regions in one chain differing in microstructure: those formed with *R*-**Ini** * and ones formed with *S*-Ini* active species

**Figure 10:**
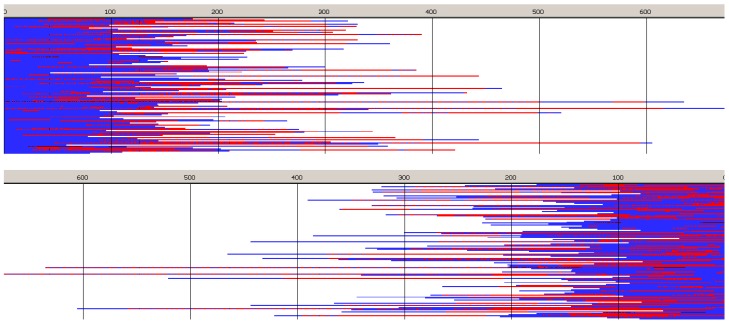
Sets of simulated copolymer chains in copolymerization initiated with the mixture of *R*- and *S*-**Ini** (94:6). Reactivity ratios as given in Table 2, *z*_LLR_ = 6.92, *z*_LLS_ = 1.45, and the ratio of **Tmc** homopropagation rate constants, *k*_TTR_/*k*_TTS,_ being about 0.78. [**Tmc**]_0_ = 2, [**Lac**]_0_ = 1.2 mol·L^−1^, and [**Ini**]_0_ = 5 × 10^−3^ (+[^i^PrOH]_0_ = 1 × 10^−2^, due to in situ synthesis of **Ini**), Conversion 95%, *DP*_n_ = 202.1, *Ð* = 1.27, average number of homoblocks in a chain equal to 25.2.

**Figure 11:**
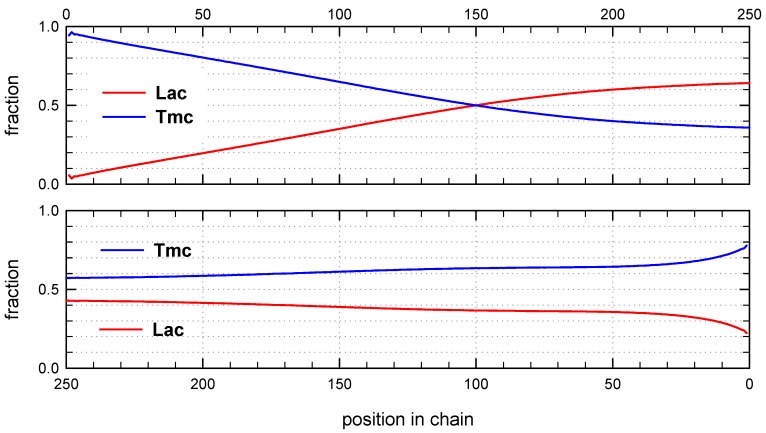
Distribution of copolymer units along chain for conditions given in Figure 10. Chain positions numerated from chain beginning (**top**) and from active center (**bottom**).

Monte Carlo simulations, presented in Figure 10 and Figure 11 (and those in Supporting Information) confirm the described briefly structure of the copolymer. The average number of blocks is significantly higher (25.3) than estimated for copolymerization initiated with *R*-**Ini** (5.2). Unfortunately, due to insufficiently fast exchange of *R* and *S* active species one can easily find (Figure 10) segments of copolymer chains containing a statistical distribution of copolymer chains. Consequently, dispersity of block-lengths is high at any chain position (see Supporting Information), being the highest at the beginning of chains (about 10 for **Lac**, and about 16 for **Tmc** blocks) and the lowest at chain positions close to active species (about four-five for both types of blocks). In the Supporting Information one can find also the plots presenting the computed distribution of dyads and the average lengths of homoblocks along chain and the discussion concerning the average homoblock lengths along copolymer chains. The average homoblock lengths can be calculated not only in dependence on chain position, but also on the way the homoblocks are selected for computing their average *DP*.

Analyzing this copolymerization and performing fitting computations, we got to a rather unexpected result. Namely, the reasonably good fitting of kinetic parameters for the chain-end *R*/*S* exchanging systems was achieved only when we have removed restriction of equal **Tmc** homopropagation rate constants on active species terminated with initiator residue **Res** of different configuration. It can be explained by solvation of **Tmc**-**Res** * active species (of both **Ini** configurations) by asymmetric **Lac** comonomer molecules and corresponding copolymer units. Depending on configuration of **Res** presumably different average numbers of asymmetric **Lac** monomers/copolymer units solvate active species, and consequently also different numbers of **Tmc** molecules can be present in the corresponding solvation spheres. Thus, different spatial molecular arrangements can be expected around **Tmc**-*R*-**Res** * and **Tmc**-*S*-**Res** * active species. In fact, taking into account asymmetric solvating entities one can consider these active species with their solvation spheres as different environments or arrangements, what results in their different reactivities, and consequently different **Tmc** homopropagation rate constants *k*_TTR_ ≠ *k*_TTS_. These differences in solvation spheres can be of two kinds: **Tmc**-*R*-**Res** * (solvated) and **Tmc**-*S*-**Res** * (solvated) can be diastereomeric if the same numbers of **Lac** monomer molecules and copolymer units are present in them, or they can differ in numbers of **Tmc** molecules if enantiomeric **Tmc**-**Res** * active species differ in accepting in their solvation spheres asymmetric **Lac** molecules, competing with symmetric **Tmc** molecules.

**Figure 12:**
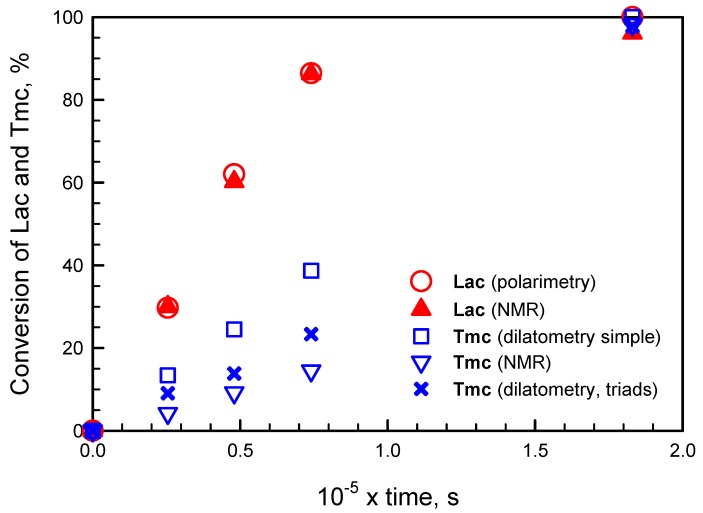
Evolution of **Lac** and **Tmc** conversions in system initiated by mixture of *R*- and *S*-**Ini** (conditions given in Figure 10) determined by ^13^C NMR, polarimetry, and two dilatometric methods: a simple (assuming equal volume contraction coefficients) and devised by us taking into account triad dependence of CC (and assuming CC_TTL_ + CC_LTT_ = CC_TTT_ + CC_LTL_, as well as *k*_TTR_ ≠ *k*_TTS_).

We believe that this presumption based on our simulations (that *k*_TTR_ ≠ *k*_TTS_) is sound because not only the corresponding fitting to experimental [**Lac**] and Δ*H* is the best but also it gives the closest agreement with experimental [**Tmc**], determined with ^13^C NMR (Figure 12).

Although this correlation between [**Tmc]**_exp_ and [**Tmc**]_calc_ obtained applying the devised method with triad dependence of volume contraction coefficients CC is not very good, we think that the observed differences stem from our approximations concerning the assumed model of copolymerization. Namely, the assumed very similar, parallel changes of rate coefficients with conversion and also the approximations concerning volume contraction coefficients may be responsible for the observed discrepancy. We think that the largest errors in estimation of **Tmc** concentrations are introduced by the limitation of the number of CC coefficients to be fitted, done by assumption that CC_AAB_ + CC_BAA_ = CC_AAA_ + CC_BAB_ (see Supplementary Materials).

## 4. Conclusions

We have shown that a bulky asymmetric initiator 2,2′-[1,1′-binaphtyl-2,2′-diyl-bis-(nitrylomethilidyne)]diphenoxy aluminum isopropoxide used in copolymerization of asymmetric monomer **Lac** with symmetric comonomer **Tmc** gives an opportunity to synthesize a product with a range of possible structures. Copolymer structure can be controlled by the choice of the initiator enantiomer, or proportion of both used simultaneously, as well as by the proportion of initial comonomer concentrations. When *R-*enantiomer is used a copolymer built of long homoblocks is formed. Moreover, it can contain some fractions of homo-**Lac** and homo-**Tmc** polymers. On the other hand, using the *S*-**Ini** results in a statistical copolymer containing random fragments with some tendency to alternacy at the beginning of chains, with approximately 1:1 distribution of copolymer units and homoblocks of **Tmc** at the end of chains (if, as in our experiments, this comonomer is used in excess).

When copolymerization is initiated with the mixture of *R*-**Ini** and *S*-**Ini** then copolymer of some intermediate structure can be obtained. For instance using the 94:6 proportion of initiator enantiomers one can obtain a multiblock copolymer with blocks much shorter than those formed while using *R* enantiomer.

Another important result of our investigation is a rather general observation that analysis of dilatometric data for copolymers may require, as it was in our systems, taking into account the different volume contraction coefficients for copolymer units in different triads. We have proposed the way of solving this analytical problem by numerical fitting of the simulated copolymerization kinetics to experimental data.

The analysis of copolymerization kinetics suggests that kinetic rate coefficients in our systems change with conversion, what was explained by variation of solvation effect involving asymmetric comonomer molecules and copolymer units. This solvation effect can also explain the difference of rate coefficients of **Tmc** homopropagation on *R-* and *S-*enantiomeric **Tmc** * active species.

## Figures and Tables

**Table 1:** Assignment of the resonance lines in the ^13^C NMR spectra of **Lac** (composed of 2 lactic units denoted here as L: **Lac** = LL) and **Tmc** copolymer units in copolymers.

	Resonance line (*i*)	Comonomer sequence	δ (ppm) ^a^
The carbonyl carbon atoms absorption range			
**Lac** repeating units (LL)	1	TLLT + LLLLT + TLLLT	- (170.24)
	2	TLT	170.14 (170.09)
	3	TLLT + TLLLL + TLLLT + LLLLT	169.97 (169.92)
	4	TLLLT + TLLLL	169.79 (169.75)
	5	LLLLL	169.61 (169.57)
**Tmc** repeating units (T)	6	TTT + LLTT + TLTT	154.89 (154.85)
	7	TTLL + LLTLL + TLTLL	154.33 (154.29)
	8	TTLT + LLTLT + TLTLT	- (153.85)
The methine carbon atoms absorption range			
**Lac** repeating units (LL)	9	TLT	- (71.98)
	10	TLLLT	71.75 (71.71)
	11	TLLT	71.65 (71.60)
	12	TLLLT + TLLLT	71.38 (71.33)
	13	TLLLL + LLLLT	69.26 (69.20)
	14	LLLLLL	69.03 (68.98)
The methylene carbon atoms absorption range ^b^			
**Tmc** repeating units (T)	15	LT′T + TT″L	64.74 (64.70)
	16	TT′T + TT″T	64.28 (64.24)
	17	LT′L + LT″L	- (61.79)
	18	TT′L + LT″T	61.70 (66.66)

^a^ In parentheses data from reference [23] are listed; ^b^ T′ = –OCH_2_CH_2_CH_2_–OCO–, T″ = –OCH_2_CH_2_CH_2_–OCO–.

**Table 2:** The reactivity ratios and relative rates of comonomer consumption ^a^ estimated for **Lac**/**Tmc** copolymerization systems initiated with *R*-**Ini** and *S*-**Ini**.

Initiator	*R*-Ini	*S*-Ini
*r*_L_	21.5	1.11
*r*_T_	2.5 × 10^2^	9.7 × 10^−2^
d[**Lac**]/d[**Tmc**]	4.1 × 10^−4^ (0.05)	0.17 (0.02)
	2.5 × 10^−3^ (0.15)	0.36 (0.05)
	8.9 × 10^−3^ (0.3)	0.71 (0.15)
	3.3 × 10^−2^ (0.6)	1.01 (0.3)
	0.35 (2)	1.43 (0.6)

^a^ Comonomer concentrations ratios corresponding to the calculated ratios of rates of comonomer consumption, assuming the validity of the Mayo-Lewis equation (steady state conditions), are given in parentheses (in bold data for initial ratios for analyzed copolymerizations are given).

## Supplementary Materials

The following are available online at www.mdpi.com/2073-4360/10/1/70/s1, PDF document containing additional spectra, plots, and equations, not included in the main text.
