# Supplementary material for: Effect of the Configuration of a Bulky Aluminum Initiator on the Structure of Copolymers of l,l-Lactide with Symmetric Comonomer Trimethylene Carbonate
Source: Polymers (Basel). 2018 Jan 13;10(1):70. doi: 10.3390/polym10010070 (PMC6414924; doi:10.3390/polym10010070)
Supplement: Supplementary file 1 [file polymers-10-00070-s001.pdf]

Supporting information

for

Effect of configuration of a bulky aluminum initiator on the structure of copolymers of *L,L*-lactide with symmetric comonomer trimethylene carbonate

Marta Socka,* Ryszard Szymanski,* Stanislaw Sosnowski, and Andrzej Duda†

Reference Homopolymerization Experiments.

To have some comparison between studied copolymerizations and homopolymerizations of the used comonomers we performed some relevant kinetic experiments using as initiators *R*- and *S*-**Ini**. Results in the form of the integral kinetics curves are shown in Figure S1. **Tmc** appeared to be the more reactive monomer for both initiator enantiomers. The apparent initial rate coefficients for homopolymerization of this monomer are, as expected, identical (in the range of possible experimental error): about $5.4\ 10^{-1}$ and $6.0\ 10^{-1}$ (±10%) L mol^{-1} s^{-1}, for polymerizations initiated with *R*-**Ini** and *S*-**Ini**, respectively. At higher conversions we see slightly higher rates of copolymerization in system with *S*-**Ini**, what we attribute to some impurities, destroying some of active species in *R*-**Ini** system. On the other hand, significant differences are observed in homopolymerizations of **Lac**, as already reported by Florczak and Duda (Angew. Chem. Int. Ed. 47 (2008) 9088–9091). Our kinetic experiments agree with the determined by the cited authors the initial rate coefficients to be $3.4\ 10^{-4}$ and $2.1\ 10^{-4}$ mol^{-1} L s^{-1}, for *R*- and *S*-**Ini** systems, respectively. Thus, the ratio of homopolymerization rate coefficients (**Tmc**/**Lac**) was estimated to be about $1.6\ 10^{3}$ and $2.9\ 10^{3}$, for *R*- and *S*-**Ini** systems, respectively.

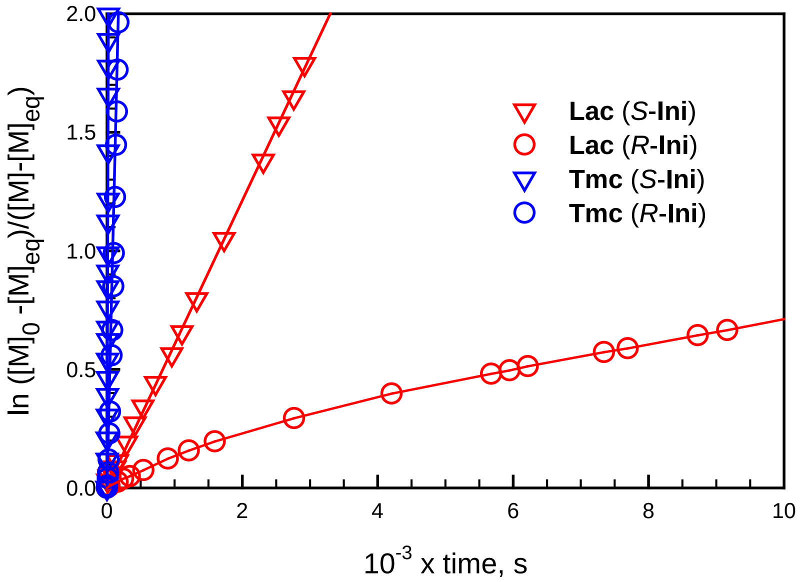

Figure S1. Semi-logarithmic kinetic plot for homopolymerization of **Tmc** and **Lac** ((*S*,*S*)-LA) in the presence of *R*-**Ini** or *S*-**Ini**. Polymerization conditions: $[R\text{-}\mathbf{Ini}]_0 = 4\ 10^{-4}$ mol L^{-1} (+ $[^{i}PrOH]_0 = 8\ 10^{-4}$ mol L^{-1} due to *in situ* synthesis of **Ini**); $[S\text{-}\mathbf{Ini}]_0 = 2\ 10^{-3}$ mol L^{-1} (+ $[^{i}PrOH]_0 = 4\ 10^{-3}$ mol L^{-1} due to *in situ* synthesis of **Ini**); $[\mathbf{Tmc}]_0 = 2.0$ mol L^{-1}, $[\mathbf{Lac}]_0 = 1.2$ mol L^{-1}, THF, 80 °C (homopolymerization equilibrium concentrations: $[\mathbf{Tmc}]_{eq} = 1.9\ 10^{-2}$ mol L^{-1}, $[\mathbf{Lac}]_{eq} = 5.5\ 10^{-2}$ mol L^{-1}).

Fitting of Results Of Numerical Simulations to Experimental Data ([Lac], Δ*H*).

Assuming validity of differential kinetic equations with constant (invariable) kinetic parameters, and negligible (from the point of view of copolymerization kinetics) change of the reaction system volume, we could compute, numerically integrating the formulated differential equations, the evolution of comonomer and various triad concentrations for any initial conditions and the assumed rate coefficient. We were using Matlab v. 7.10 environment and Matlab function *ode15s*, for integration of differential equations (stiff systems). Next, assuming the volume contraction coefficients valid for copolymer units of the central triad positions, we could compute the small change of the system volume and, taking into account the volume of dilatometer and diameter of capillary, the change of meniscus height ΔH.

In order to relate ΔH with copolymerization conversion we have assumed that the average molar volume of a copolymer unit A (A = **Lac** and/or **Tmc**), $V_{M(copo)}$, depends on copolymer composition and on its microstructure (mole fractions of **Lac** and **Tmc** units and of corresponding triads), and different molar volumes of a unit in different triads:

$$V_{A(copo)}=(x_{AAA}\cdot V_{AAA} + x_{AAB}\cdot V_{AAB} + x_{BAA}\cdot V_{BAA} + x_{BAB}\cdot V_{BAB})/x_A \quad (S1)$$

(x denote molar fractions of the corresponding unit or triad, assuming sufficiently long chains we have $x_A = x_{AAA} + x_{AAB} + x_{BAA} + x_{BAB}$)

The molar change of volume due to copolymerization of A (A, B = **Lac** or **Tmc**, A ≠ B, is equal to the corresponding difference of molar volumes of the average copolymer unit and corresponding comonomer:

$$\Delta V_{A(copo)} = V_{A(copo)} - V_{A(monomer)} = (x_{AAA}\cdot \Delta V_{AAA} + x_{AAB}\cdot \Delta V_{AAB} + x_{BAA}\cdot \Delta V_{BAA} + x_{BAB}\cdot \Delta V_{BAB})/x_A \quad (S2)$$

These molar changes of volume, corresponding to various triads, are regarded by us as the volume contraction coefficients CC: $CC_{TTL} = \Delta V_{TTL}$, etc., where L and T refer to **Lac** and **Tmc** units, respectively, located in the indicated triad (CC describes the change of molar volume of the unit in the triad central location).

In order to limit the number of fitted parameters we additionally assumed that CC of homotriads are equal to the corresponding CC of polymer units in the corresponding homopolymer and thus determined experimentally: $CC_{LLL} = CC_{L(homo)} = 0$ (no change of volume in **Lac** homopolymerization), $CC_{TTT} = CC_{T(homo)} = 1.51\ 10^{-3}$ L mol^{-1}.

Moreover, we also assumed that CC for asymmetric triads (CC_{LLT} and CC_{TLL}, as well as CC_{LTT} and CC_{TTL}) do not have to be separately regarded because in sufficiently long chains the numbers of the corresponding triads with the same central unit are equal, unless the product is a diblock, or with a small number of blocks, copolymer. When chains are sufficiently long the cases of copolymers with a low number of blocks can also be treated in this simplified way, because the possible error in the change of volume of copolymerizing system is negligible (contribution of such triads is much lower than of homotriads).

Moreover, for the sake of limiting the number or fitted parameters, we applied an arbitrary approximation that the average change of the molar volumes of asymmetric triads equals to the average change of molar volumes of the corresponding homo and alternate triads:

$$(\Delta V_{AAB} + \Delta V_{BAA})/2 = (\Delta V_{AAA} + \Delta V_{BAB})/2 = CC_{A(hetero)} \quad (S3)$$

Finally, we got to the approximate equations relating the change of the meniscus height ΔH with the concentrations of various triads in copolymerizing system.

$$\Delta V_{L(copo)} = \frac{x_{LLL}CC_{L(homo)} + (x_{LLT} + x_{TLL})CC_{L(hetero)} + x_{TLT}CC_{TLT}}{x_L} \quad (S4)$$

$$\Delta V_{T(copo)} = \frac{x_{TTT}CC_{T(homo)} + (x_{TTL} + x_{LTT})CC_{T(hetero)} + x_{LTL}CC_{LTL}}{x_T} \quad (S5)$$

$$\begin{aligned}\Delta V_{M(copo)} &= x_L \Delta V_{L(copo)} + x_T \Delta V_{T(copo)} \\ &= x_{LLL} CC_{L(homo)} + \left(x_{LLT} + x_{TLL}\right) CC_{L(hetero)} + x_{TLT} CC_{TLT} \\ &\quad + x_{TTT} CC_{T(homo)} + \left(x_{TTL} + x_{LTT}\right) CC_{T(hetero)} + x_{LTL} CC_{LTL}\end{aligned} \quad (S6)$$

$$\begin{aligned}\Delta Vol_{(copo)} &= \left([-L-]+[-T-]\right) \times Vol \times V_{M(copo)} = \\ &= \begin{Bmatrix} CC_{L(homo)}[LLL] + \left([LLT] + CC_{L(hetero)}[TLL]\right) + CC_{TLT}[TLT] \\ + CC_{T(homo)}[TTT] + CC_{T(hetero)}\left([TTL]+[LTT]\right) + CC_{LTL}[LTL] \end{Bmatrix} \times Vol\end{aligned} \quad (S7)$$

$$\Delta H = \frac{\Delta Vol_{(copo)}}{\pi d^2/4} = \begin{Bmatrix} CC_{L(homo)}[LLL] + \left([LLT] + CC_{L(hetero)}[TLL]\right) + CC_{TLT}[TLT] \\ + CC_{T(homo)}[TTT] + CC_{T(hetero)}\left([TTL]+[LTT]\right) + CC_{LTL}[LTL] \end{Bmatrix} \times \frac{4}{\pi} \quad (S8)$$

$\Delta V_{X(copo)}$ is the molar volume change due to copolymerization of comonomer X = L, T, and M, denoting **Lac**, **Tmc**, and both, respectively, $\Delta Vol_{(copo)}$ is the change of the copolymerizing system and Vol is the initial volume of this system, while *d* is the diameter of the dilatometer capillary.

Thus, when the considered differential equations were formulated in time scale, we directly obtained the computed concentrations of comonomers and all triads for any experimental reaction time, and consequently, applied the derived above equations to ΔH values as well. Fitting of kinetic parameters and of the volume contraction coefficients could be done easily using the Matlab function *fminsearch*, starting from various initial values of the considered parameters. The objective function *D* being minimized was the sum of absolute values of normalized differences between the calculated and experimental [**Lac**] and ΔH values (vectors of corresponding data related to the vector of experimental times):

$$D = sum\left(abs\left([\mathbf{Lac}]_{calc} - [\mathbf{Lac}]_{exp}\right)/[\mathbf{Lac}]_0\right) + sum\left(abs\left(\Delta H_{calc} - \Delta H_{exp}\right)/\max(\Delta H_{exp})\right) \quad (S9)$$

However, when the differential equations in conversion scale were being integrated, we have chosen arbitrarily the conversion scale containing 100 values: 0, 0.01, 0.02, ... 0.99, and we computed the [**Lac**] and ΔH values correspondingly. In order to fit kinetic and contraction coefficients to experimental data we performed next recalculation of [**Lac**] and ΔH values for the reaction times, assuming that the auxiliary monotonously decreasing variable $u = [\mathbf{Lac}]/[\mathbf{Lac}]_0 - \Delta H/\max(\Delta H)$, formulated for both experimental and calculated [**Lac**] and ΔH values (u_{exp}, u_{calc}), gives access to the needed relationship. It is so, because only if fitting is satisfactory the functions $\{[\mathbf{Lac}]_{exp}, \Delta H_{exp}\} = f(u_{exp})$ and $\{[\mathbf{Lac}]_{calc}, \Delta H_{calc}\} = f(u_{calc})$ present the same relationships. The required simulated values of $[\mathbf{Lac}]_{time}$ and ΔH_{time} (calculated values corresponding to the given reaction times) were obtained applying numerical interpolation, applying the Matlab function *interp1*:

$$\begin{aligned}[\mathbf{Lac}]_{time} &= interp1\left(u_{calc}, [\mathbf{Lac}]_{calc}, u_{exp}, 'pchip', 'extrap'\right) \\ \Delta H_{time} &= interp1\left(u_{calc}, \Delta H_{calc}, u_{exp}, 'pchip', 'extrap'\right)\end{aligned} \quad (S10)$$

Parameter fitting was performed as previously, applying the Matlab function *fminsearch*. The objective function being minimized was defined as previously:

$$D = sum\left(abs\left([\mathbf{Lac}]_{time} - [\mathbf{Lac}]_{exp}\right)/[\mathbf{Lac}]_0\right) + sum\left(abs\left(\Delta H_{time} - \Delta H_{exp}\right)/\max(\Delta H_{exp})\right) \quad (S11)\ (1)$$

Differential equations describing Lac/Tmc copolymerization, assuming fast exchange of active and OH terminated copolymer chains and independence of rate coefficients of conversion. Volume changes were assumed to be negligible for kinetics of copolymerization.

Equations formulated for time scale:

$$\begin{aligned}
&\frac{d[\mathrm{L}^*]}{dt} = -\frac{d[\mathrm{T}^*]}{dt} = -\mathrm{k}_{\mathrm{LT}}[\mathrm{L}^*][\mathrm{T}] + \mathrm{k}_{\mathrm{TL}}[\mathrm{T}^*][\mathrm{L}] \\
&\frac{d[\mathrm{L}]}{dt} = -\mathrm{k}_{\mathrm{LL}}[\mathrm{L}^*][\mathrm{L}] - \mathrm{k}_{\mathrm{TL}}[\mathrm{T}^*][\mathrm{L}] \\
&\frac{d[\mathrm{T}]}{dt} = -\mathrm{k}_{\mathrm{TT}}[\mathrm{T}^*][\mathrm{T}] - \mathrm{k}_{\mathrm{LT}}[\mathrm{L}^*][\mathrm{T}] \\
&\frac{d[\mathrm{LL}^*]}{dt} = \mathrm{k}_{\mathrm{LL}}\left([\mathrm{L}^*] - [\mathrm{LL}^*]\right)[\mathrm{L}] - \mathrm{k}_{\mathrm{LT}}[\mathrm{LL}^*][\mathrm{T}] \\
&\frac{d[\mathrm{TL}^*]}{dt} = \mathrm{k}_{\mathrm{TL}}[\mathrm{T}^*][\mathrm{L}] - \mathrm{k}_{\mathrm{LL}}[\mathrm{TL}^*][\mathrm{L}] - \mathrm{k}_{\mathrm{LT}}[\mathrm{TL}^*][\mathrm{T}]\text{ , etc.} \\
&\frac{d[\mathrm{LLL}]}{dt} = \mathrm{k}_{\mathrm{LL}}[\mathrm{LL}^*][\mathrm{L}] \\
&\frac{d[\mathrm{LLT}]}{dt} = \mathrm{k}_{\mathrm{LT}}[\mathrm{LL}^*][\mathrm{T}]\text{ , etc.}
\end{aligned} \tag{S12}$$

These equations are valid independently of that if the rate coefficients are constant (invariable with conversion) or changing during copolymerization. However, while performing integration of the differential equations for systems with rate coefficients dependent on reaction time one would need knowing the corresponding functions ($\mathrm{k}_{\mathrm{reaction}} = \mathrm{f}(t)$).

We have assumed that these time functions are similar, resulting in $\mathrm{k}_{\mathrm{reaction1}}/\mathrm{k}_{\mathrm{reaction2}}$ being invariable. This assumption led us to formulation of the differential equations in conversion scale with relative rate parameters independent of conversion.

Equations formulated for conversion scale (S13):

$$\frac{d[\mathrm{L}^*]}{dConv}=-\frac{d[\mathrm{T}^*]}{dConv}=\left([\mathrm{L}]_0+[\mathrm{B}]_0\right)\times\frac{-\frac{z_{LL}}{r_L}[\mathrm{L}^*][\mathrm{T}]+\frac{1}{r_T}[\mathrm{T}^*][\mathrm{L}]}{z_{LL}[\mathrm{L}^*][\mathrm{L}]+\frac{1}{r_T}[\mathrm{T}^*][\mathrm{L}]+[\mathrm{T}^*][\mathrm{T}]+\frac{z_{LL}}{r_L}[\mathrm{L}^*][\mathrm{T}]}$$

$$\frac{d[\mathrm{L}]}{dConv}=-\left([\mathrm{L}]_0+[\mathrm{T}]_0\right)\times\frac{z_{LL}[\mathrm{L}^*][\mathrm{L}]+\frac{1}{r_T}[\mathrm{T}^*][\mathrm{L}]}{z_{LL}[\mathrm{L}^*][\mathrm{L}]+\frac{1}{r_T}[\mathrm{T}^*][\mathrm{L}]+[\mathrm{T}^*][\mathrm{T}]+\frac{z_{LL}}{r_L}[\mathrm{L}^*][\mathrm{T}]}$$

$$\frac{d[\mathrm{T}]}{dConv}=-\left([\mathrm{L}]_0+[\mathrm{T}]_0\right)\times\frac{[\mathrm{T}^*][\mathrm{T}]+\frac{z_{LL}}{r_L}[\mathrm{L}^*][\mathrm{T}]}{z_{LL}[\mathrm{L}^*][\mathrm{L}]+\frac{1}{r_T}[\mathrm{T}^*][\mathrm{L}]+[\mathrm{T}^*][\mathrm{T}]+\frac{z_{LL}}{r_L}[\mathrm{L}^*][\mathrm{T}]}$$

$$\frac{d[\mathrm{LL}^*]}{dConv}=\left([\mathrm{L}]_0+[\mathrm{T}]_0\right)\times\frac{z_{LL}\left([\mathrm{L}^*]-[\mathrm{LL}^*]\right)[\mathrm{L}]-\frac{z_{LL}}{r_L}[\mathrm{LL}^*][\mathrm{T}]}{z_{LL}[\mathrm{L}^*][\mathrm{L}]+\frac{1}{r_T}[\mathrm{T}^*][\mathrm{L}]+[\mathrm{T}^*][\mathrm{T}]+\frac{z_{LL}}{r_L}[\mathrm{L}^*][\mathrm{T}]} \qquad \text{(S13)}$$

$$\frac{d[\mathrm{TL}^*]}{dConv}=\left([\mathrm{L}]_0+[\mathrm{T}]_0\right)\times\frac{\frac{1}{r_T}[\mathrm{T}^*][\mathrm{L}]-z_{LL}[\mathrm{TL}^*][\mathrm{L}]-\frac{z_{LL}}{r_L}[\mathrm{TL}^*][\mathrm{T}]}{z_{LL}[\mathrm{L}^*][\mathrm{L}]+\frac{1}{r_T}[\mathrm{T}^*][\mathrm{L}]+[\mathrm{T}^*][\mathrm{T}]+\frac{z_{LL}}{r_L}[\mathrm{L}^*][\mathrm{T}]}\text{, etc.}$$

$$\frac{d[\mathrm{LLL}]}{dConv}=\left([\mathrm{L}]_0+[\mathrm{T}]_0\right)\times\frac{z_{LL}[\mathrm{LL}^*][\mathrm{L}]}{z_{L}[\mathrm{L}^*][\mathrm{L}]+\frac{1}{r_T}[\mathrm{T}^*][\mathrm{L}]+[\mathrm{T}^*][\mathrm{T}]+\frac{z_{LL}}{r_L}[\mathrm{L}^*][\mathrm{T}]}$$

$$\frac{d[\mathrm{LLB}]}{dConv}=\left([\mathrm{L}]_0+[\mathrm{B}]_0\right)\times\frac{\frac{z_{LL}}{r_L}[\mathrm{LL}^*][\mathrm{B}]}{z_{LL}[\mathrm{L}^*][\mathrm{L}]+\frac{1}{r_B}[\mathrm{B}^*][\mathrm{L}]+[\mathrm{B}^*][\mathrm{B}]+\frac{z_{LL}}{r_L}[\mathrm{L}^*][\mathrm{B}]}\text{, etc.}$$

Differential equations describing Lac/Tmc copolymerization, taking into account the exchange of active and OH terminated copolymer chains and independence of rate coefficients of conversion. Volume changes were assumed to be negligible for kinetics of copolymerization.

Equation set formulated for the time scale, taking into account the rates of exchange of active and OH chain-ends, differ from the one given above (assuming the sufficiently fast exchanging active and OH bearing chains) only with different equations for active species and additional equations for OH terminated chains. Consequently, the equations for comonomer and triad concentrations, being identical as previously, are omitted below:

$$
\begin{aligned}
&\frac{d[\mathrm{L}^*]}{dt}=-\frac{d[\mathrm{T}^*]}{dt}=-\mathrm{k}_{\mathrm{LT}}[\mathrm{L}^*][\mathrm{T}]+\mathrm{k}_{\mathrm{TL}}[\mathrm{T}^*][\mathrm{L}]+\mathrm{k}_{\mathrm{LOHT}^*}[\mathrm{LOH}][\mathrm{T}^*]-\mathrm{k}_{\mathrm{TOHL}^*}[\mathrm{TOH}][\mathrm{L}^*]\\
&\frac{d[\mathrm{LL}^*]}{dt}=\mathrm{k}_{\mathrm{LL}}\left([\mathrm{L}^*]-[\mathrm{LL}^*]\right)[\mathrm{L}]-\mathrm{k}_{\mathrm{LT}}[\mathrm{LL}^*][\mathrm{T}]+\mathrm{k}_{\mathrm{LOHL}^*}\left([\mathrm{L}^*]-[\mathrm{LL}^*]\right)[\mathrm{LLOH}]+\\
&\qquad \mathrm{k}_{\mathrm{LOHT}^*}[\mathrm{LLOH}][\mathrm{T}^*]-\mathrm{k}_{\mathrm{TOHL}^*}[\mathrm{TOH}][\mathrm{LL}^*]-\mathrm{k}_{\mathrm{LOHL}^*}\left([\mathrm{LOH}]-[\mathrm{LLOH}]\right)[\mathrm{LL}^*]\\
&\frac{d[\mathrm{TL}^*]}{dt}=\mathrm{k}_{\mathrm{TL}}[\mathrm{T}^*][\mathrm{L}]-\mathrm{k}_{\mathrm{LL}}[\mathrm{TL}^*][\mathrm{L}]-\mathrm{k}_{\mathrm{LT}}[\mathrm{TL}^*][\mathrm{T}]+\mathrm{k}_{\mathrm{LOHL}^*}[\mathrm{TLOH}]\left([\mathrm{L}^*]-[\mathrm{TL}^*]\right)+\\
&\qquad \mathrm{k}_{\mathrm{LOHT}^*}[\mathrm{TLOH}][\mathrm{T}^*]-\mathrm{k}_{\mathrm{TOHL}^*}[\mathrm{TOH}][\mathrm{TL}^*]-\mathrm{k}_{\mathrm{LOHL}^*}\left([\mathrm{LOH}]-[\mathrm{TLOH}]\right)[\mathrm{TL}^*]\text{, etc.}\\
&\frac{d[\mathrm{LOH}]}{dt}=-\frac{d[\mathrm{TOH}]}{dt}=-\mathrm{k}_{\mathrm{LOHT}^*}[\mathrm{LOH}][\mathrm{T}^*]+\mathrm{k}_{\mathrm{TOHL}^*}[\mathrm{TOH}][\mathrm{L}^*] \qquad \text{(S14)}\\
&\frac{d[\mathrm{LLOH}]}{dt}=\mathrm{k}_{\mathrm{TOHL}^*}[\mathrm{TOH}][\mathrm{LL}^*]-\mathrm{k}_{\mathrm{LOHT}^*}[\mathrm{LLOH}][\mathrm{T}^*]-\mathrm{k}_{\mathrm{LOHL}^*}\left([\mathrm{L}^*]-[\mathrm{LL}^*]\right)[\mathrm{LLOH}]+\\
&\qquad \mathrm{k}_{\mathrm{LOHL}^*}\left([\mathrm{LOH}]-[\mathrm{LLOH}]\right)[\mathrm{LL}^*]\\
&\qquad =\mathrm{k}_{\mathrm{TOHL}^*}[\mathrm{TOH}][\mathrm{LL}^*]-\mathrm{k}_{\mathrm{LOHT}^*}[\mathrm{LLOH}][\mathrm{T}^*]+\mathrm{k}_{\mathrm{LOHL}^*}\left([\mathrm{LOH}][\mathrm{LL}^*]-[\mathrm{L}^*][\mathrm{LLOH}]\right)\\
&\frac{d[\mathrm{TLOH}]}{dt}=\mathrm{k}_{\mathrm{TOHL}^*}[\mathrm{TOH}][\mathrm{TL}^*]-\mathrm{k}_{\mathrm{LOHT}^*}[\mathrm{TLOH}][\mathrm{T}^*]+\mathrm{k}_{\mathrm{LOHL}^*}\left([\mathrm{LOH}]-[\mathrm{TLOH}]\right)[\mathrm{TL}^*]+\\
&\qquad -\mathrm{k}_{\mathrm{LOHL}^*}[\mathrm{TLOH}]\left([\mathrm{L}^*]-[\mathrm{TL}^*]\right)\\
&\qquad =\mathrm{k}_{\mathrm{TOHL}^*}[\mathrm{TOH}][\mathrm{TL}^*]-\mathrm{k}_{\mathrm{LOHT}^*}[\mathrm{TLOH}][\mathrm{T}^*]+\mathrm{k}_{\mathrm{LOHL}^*}\left([\mathrm{LOH}][\mathrm{TL}^*]-[\mathrm{TLOH}][\mathrm{L}^*]\right)\text{, etc.}
\end{aligned}
$$

Similarly, as previously, the differential equations in conversion scale, with constant relative rate coefficients were formulated. For the sake of simplicity, the approximation was done, assuming all chain-end exchange coefficients being equal one to another, $k_{XOHY^*} = k_{ex}$. The corresponding relative rate coefficient was denoted as z_{ex}, equal to k_{ex}/k_{TT}.

Consequently, the kinetic differential equations in conversion scale were formulated. The ones, corresponding to the equation set (S15) are given below.

$$\frac{d[L^*]}{dConv}=-\frac{d[T^*]}{dConv}=([L]_0+[B]_0)\times\frac{-\frac{z_{LL}}{r_L}[L^*][T]+\frac{1}{r_T}[T^*][L]+z_{ex}([LOH][T^*]-[TOH][L^*])}{z_{LL}[L^*][L]+\frac{1}{r_T}[T^*][L]+[T^*][T]+\frac{z_{LL}}{r_L}[L^*][T]}$$

$$\frac{d[LL^*]}{dConv}=([L]_0+[T]_0)\times\left(\frac{z_{LL}([L^*]-[LL^*])[L]-\frac{z_{LL}}{r_L}[LL^*][T]+z_{ex}[LLOH][T^*]}{z_{LL}[L^*][L]+\frac{1}{r_T}[T^*][L]+[T^*][T]+\frac{z_{LL}}{r_L}[L^*][T]}+\frac{z_{ex}\{([L^*]-[LL^*])[LLOH]-([LOH]-[LLOH])[LL^*]-[TOH][LL^*]\}}{z_{LL}[L^*][L]+\frac{1}{r_T}[T^*][L]+[T^*][T]+\frac{z_{LL}}{r_L}[L^*][T]}\right)$$

$$\frac{d[TL^*]}{dConv}=([L]_0+[T]_0)\times\left(\frac{\frac{1}{r_T}[T^*][L]-z_{LL}[TL^*][L]-\frac{z_{LL}}{r_L}[TL^*][T]+z_{ex}[TLOH][T^*]}{z_{LL}[L^*][L]+\frac{1}{r_T}[T^*][L]+[T^*][T]+\frac{z_{LL}}{r_L}[L^*][T]}+\frac{z_{ex}\{[TLOH]([L^*]-[TL^*])-([LOH]-[TLOH])[TL^*]-[TOH][TL^*]\}}{z_{LL}[L^*][L]+\frac{1}{r_T}[T^*][L]+[T^*][T]+\frac{z_{LL}}{r_L}[L^*][T]}\right)\text{, etc.}\quad\text{(S15)}$$

$$\frac{d[LOH]}{dConv}=-\frac{d[TOH]}{dConv}=([L]_0+[B]_0)\times\frac{z_{ex}([TOH][L^*]-[LOH][T^*])}{z_{LL}[L^*][L]+\frac{1}{r_T}[T^*][L]+[T^*][T]+\frac{z_{LL}}{r_L}[L^*][T]}$$

$$\frac{d[LLOH]}{dConv}=([L]_0+[B]_0)\times\frac{z_{ex}\{[TOH][LL^*]-[LLOH][T^*]+([LOH][LL^*]-[L^*][LLOH])\}}{z_{LL}[L^*][L]+\frac{1}{r_T}[T^*][L]+[T^*][T]+\frac{z_{LL}}{r_L}[L^*][T]}$$

$$\frac{d[TLOH]}{dConv}=([L]_0+[B]_0)\times\frac{z_{ex}\{[TOH][TL^*]-[TLOH][T^*]+([LOH][TL^*]-[TLOH][L^*])\}}{z_{LL}[L^*][L]+\frac{1}{r_T}[T^*][L]+[T^*][T]+\frac{z_{LL}}{r_L}[L^*][T]}\text{, etc.}$$

Differential equations describing Lac/Tmc copolymerization, taking into account the exchange of active and OH terminated copolymer chains and independence of rate coefficients of conversion. Initiation with the mixture of Ini enantiomers. Volume changes were assumed to be negligible for kinetics of copolymerization.

Differential equations in conversion scale are given below.

$$
\begin{aligned}
DN ={}& z_{\mathrm{LLR}}[\mathrm{LR^*}][\mathrm{L}]+\frac{1}{r_{\mathrm{TR}}}[\mathrm{TR^*}][\mathrm{L}]+[\mathrm{TR^*}][\mathrm{T}]+\frac{z_{\mathrm{LLR}}}{r_{\mathrm{LR}}}[\mathrm{LR^*}][\mathrm{T}] \\
&+p_{\mathrm{RS}}\left(z_{\mathrm{LLS}}[\mathrm{LS^*}][\mathrm{L}]+\frac{1}{r_{\mathrm{TS}}}[\mathrm{TS^*}][\mathrm{L}]+[\mathrm{TS^*}][\mathrm{T}]+\frac{z_{\mathrm{LLS}}}{r_{\mathrm{LS}}}[\mathrm{LS^*}][\mathrm{T}]\right) \\
\frac{d[\mathrm{L}]}{dConv} ={}& -\left([\mathrm{L}]_0+[\mathrm{T}]_0\right)\times\frac{z_{\mathrm{LLR}}[\mathrm{LR^*}][\mathrm{L}]+\frac{1}{r_{\mathrm{TR}}}[\mathrm{TR^*}][\mathrm{L}]+p_{\mathrm{RS}}\left(z_{\mathrm{LLS}}[\mathrm{LS^*}][\mathrm{L}]+\frac{1}{r_{\mathrm{TS}}}[\mathrm{TS^*}][\mathrm{L}]\right)}{DN} \\
\frac{d[\mathrm{T}]}{dConv} ={}& -\left([\mathrm{L}]_0+[\mathrm{T}]_0\right)\times\frac{[\mathrm{TR^*}][\mathrm{T}]+\frac{z_{\mathrm{LLR}}}{r_{\mathrm{LR}}}[\mathrm{LR^*}][\mathrm{T}]+p_{\mathrm{RS}}\left([\mathrm{TS^*}][\mathrm{T}]+\frac{z_{\mathrm{LLS}}}{r_{\mathrm{LS}}}[\mathrm{LS^*}][\mathrm{T}]\right)}{DN} \\
\frac{d[\mathrm{LR^*}]}{dConv} ={}& -\frac{d[\mathrm{TR^*}]}{dConv}=\left([\mathrm{L}]_0+[\mathrm{T}]_0\right)\times\frac{-\frac{z_{\mathrm{LLR}}}{r_{\mathrm{LR}}}[\mathrm{LR^*}][\mathrm{T}]+\frac{1}{r_{\mathrm{TR}}}[\mathrm{TR^*}][\mathrm{L}]+z_{ex\mathrm{R}}\left([\mathrm{LOH}][\mathrm{TR^*}]-[\mathrm{TOH}][\mathrm{LR^*}]\right)}{DN} \\
\frac{d[\mathrm{LS^*}]}{dConv} ={}& -\frac{d[\mathrm{TS^*}]}{dConv}=\left([\mathrm{L}]_0+[\mathrm{T}]_0\right)p_{\mathrm{RS}}\times\frac{-\frac{z_{\mathrm{LLS}}}{r_{\mathrm{LS}}}[\mathrm{LS^*}][\mathrm{T}]+\frac{1}{r_{\mathrm{TS}}}[\mathrm{TS^*}][\mathrm{L}]+z_{ex\mathrm{S}}\left([\mathrm{LOH}][\mathrm{TS^*}]-[\mathrm{TOH}][\mathrm{LS^*}]\right)}{DN} \\
\frac{d[\mathrm{LLR^*}]}{dConv} ={}& \left([\mathrm{L}]_0+[\mathrm{T}]_0\right)\times\left(\begin{aligned}&\frac{z_{\mathrm{LLR}}\left([\mathrm{LR^*}]-[\mathrm{LLR^*}]\right)[\mathrm{L}]-\frac{z_{\mathrm{LLR}}}{r_{\mathrm{LR}}}[\mathrm{LLR^*}][\mathrm{T}]+z_{ex\mathrm{R}}[\mathrm{LLOH}][\mathrm{TR^*}]}{DN}+\\&\frac{z_{ex\mathrm{R}}\left\{\left([\mathrm{LR^*}]-[\mathrm{LLR^*}]\right)[\mathrm{LLOH}]-\left([\mathrm{LOH}]-[\mathrm{LLOH}]\right)[\mathrm{LLR^*}]-[\mathrm{TOH}][\mathrm{LLR^*}]\right\}}{DN}\end{aligned}\right) \qquad \text{(S16)} \\
\frac{d[\mathrm{TLR^*}]}{dConv} ={}& \left([\mathrm{L}]_0+[\mathrm{T}]_0\right)\times\left(\begin{aligned}&\frac{\frac{1}{r_{\mathrm{TR}}}[\mathrm{TR^*}][\mathrm{L}]-z_{\mathrm{LLR}}[\mathrm{TLR^*}][\mathrm{L}]-\frac{z_{\mathrm{LLR}}}{r_{\mathrm{LR}}}[\mathrm{TLR^*}][\mathrm{T}]+z_{ex\mathrm{R}}[\mathrm{TLOH}][\mathrm{TR^*}]}{DN}+\\&\frac{z_{ex\mathrm{R}}\left\{[\mathrm{TLOH}]\left([\mathrm{LR^*}]-[\mathrm{TLR^*}]\right)-\left([\mathrm{LOH}]-[\mathrm{TLOH}]\right)[\mathrm{TLR^*}]-[\mathrm{TOH}][\mathrm{TLR^*}]\right\}}{DN}\end{aligned}\right),\ \text{etc.} \\
\frac{d[\mathrm{LLS^*}]}{dConv} ={}& \left([\mathrm{L}]_0+[\mathrm{T}]_0\right)p_{\mathrm{RS}}\times\left(\begin{aligned}&\frac{z_{\mathrm{LLS}}\left([\mathrm{LS^*}]-[\mathrm{LLS^*}]\right)[\mathrm{L}]-\frac{z_{\mathrm{LLS}}}{r_{\mathrm{LS}}}[\mathrm{LLS^*}][\mathrm{T}]+z_{ex\mathrm{S}}[\mathrm{LLOH}][\mathrm{TS^*}]}{DN}+\\&\frac{z_{ex\mathrm{S}}\left\{\left([\mathrm{LS^*}]-[\mathrm{LLS^*}]\right)[\mathrm{LLOH}]-\left([\mathrm{LOH}]-[\mathrm{LLOH}]\right)[\mathrm{LLS^*}]-[\mathrm{TOH}][\mathrm{LLS^*}]\right\}}{DN}\end{aligned}\right) \\
\frac{d[\mathrm{TLS^*}]}{dConv} ={}& \left([\mathrm{L}]_0+[\mathrm{T}]_0\right)p_{\mathrm{RS}}\times\left(\begin{aligned}&\frac{\frac{1}{r_{\mathrm{TS}}}[\mathrm{TS^*}][\mathrm{L}]-z_{\mathrm{LLS}}[\mathrm{TLS^*}][\mathrm{L}]-\frac{z_{\mathrm{LLS}}}{r_{\mathrm{LS}}}[\mathrm{TLS^*}][\mathrm{T}]+z_{ex\mathrm{S}}[\mathrm{TLOH}][\mathrm{TS^*}]}{DN}+\\&\frac{z_{ex\mathrm{S}}\left\{[\mathrm{TLOH}]\left([\mathrm{LS^*}]-[\mathrm{TLS^*}]\right)-\left([\mathrm{LOH}]-[\mathrm{TLOH}]\right)[\mathrm{TLS^*}]-[\mathrm{TOH}][\mathrm{TLS^*}]\right\}}{DN}\end{aligned}\right),\ \text{etc.}
\end{aligned}
$$

$$\begin{aligned}
&\frac{d[\mathrm{LOH}]}{dConv}=-\frac{d[\mathrm{TOH}]}{dConv}=\left([\mathrm{L}]_0+[\mathrm{B}]_0\right)\times\left(\frac{z_{exR}\left([\mathrm{TOH}][\mathrm{LR^*}]-[\mathrm{LOH}][\mathrm{TR^*}]\right)}{DN}+\frac{p_{\mathrm{RS}}z_{exS}\left([\mathrm{TOH}][\mathrm{LS^*}]-[\mathrm{LOH}][\mathrm{TS^*}]\right)}{DN}\right)\\
&\frac{d[\mathrm{LLOH}]}{dConv}=\left([\mathrm{L}]_0+[\mathrm{B}]_0\right)\times\left(\frac{z_{exR}\left\{[\mathrm{TOH}][\mathrm{LLR^*}]-[\mathrm{LLOH}][\mathrm{TR^*}]+\left([\mathrm{LOH}][\mathrm{LLR^*}]-[\mathrm{LR^*}][\mathrm{LLOH}]\right)\right\}}{DN}+\frac{p_{\mathrm{RS}}z_{exS}\left\{[\mathrm{TOH}][\mathrm{LLS^*}]-[\mathrm{LLOH}][\mathrm{TS^*}]+\left([\mathrm{LOH}][\mathrm{LLS^*}]-[\mathrm{LS^*}][\mathrm{LLOH}]\right)\right\}}{DN}\right)\\
&\frac{d[\mathrm{TLOH}]}{dConv}=\left([\mathrm{L}]_0+[\mathrm{B}]_0\right)\times\left(\frac{z_{exR}\left\{[\mathrm{TOH}][\mathrm{TLR^*}]-[\mathrm{TLOH}][\mathrm{TR^*}]+\left([\mathrm{LOH}][\mathrm{TLR^*}]-[\mathrm{TLOH}][\mathrm{LR^*}]\right)\right\}}{DN}+\frac{p_{\mathrm{RS}}z_{exS}\left\{[\mathrm{TOH}][\mathrm{TLS^*}]-[\mathrm{TLOH}][\mathrm{TS^*}]+\left([\mathrm{LOH}][\mathrm{TLS^*}]-[\mathrm{TLOH}][\mathrm{LS^*}]\right)\right\}}{DN}\right)\text{, etc.}
\end{aligned} \tag{S17}$$

where 'R' and 'S' refer the enantiomer of the **Ini** residue located at active chain-end and p_{RS} is the ratio of **Tmc** homopropagation rate constants concerning enatiomeric active species **Tmc**-*R*-**Ini*** and **Tmc**-*S*-**Ini***, p_{RS} = k_{TTR}/k_{TTS}, presumably to be equal to 1, as one can expect reactivity of enantiomeric active species identical.

However, the best fitting of the relative rate coefficients was obtained assuming $k_{TTR} \neq k_{TTS}$ (see main text), what was explained with solvation sphere containing asymmetric molecules, making **Tmc**-*R*-**Ini*** and **Tmc**-*S*-**Ini*** diastereomeric.

Copolymerization initiated with the mixture of R- and S-Ini. SEC chromatograms and 13CNMR spectra.

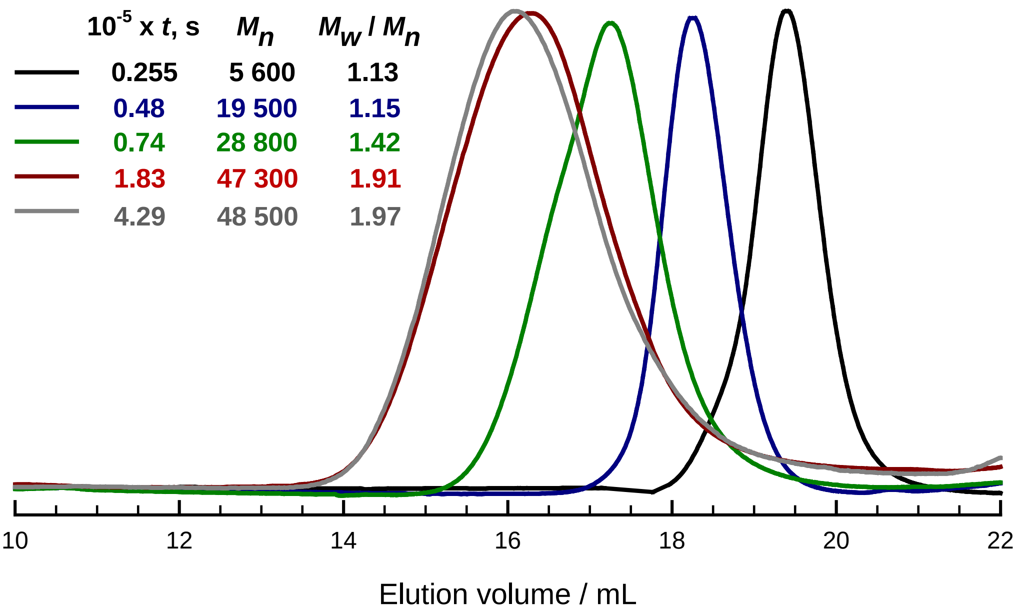

Figure S2. SEC chromatograms of copolymer obtained in copolymerization initiated with the mixture of *R*- and *S*-**Ini** (94:6). Polymerization conditions: $[\mathbf{Lac}]_0$ = 1.2, $[\mathbf{Tmc}]_0$ = 2, $[\mathbf{Ini}]_0$ = 2 10^{-3} mol L^{-1} (+ $[^{i}\mathrm{PrOH}]_0$ = 4 10^{-3} mol L^{-1} due to *in situ* synthesis of **Ini**).

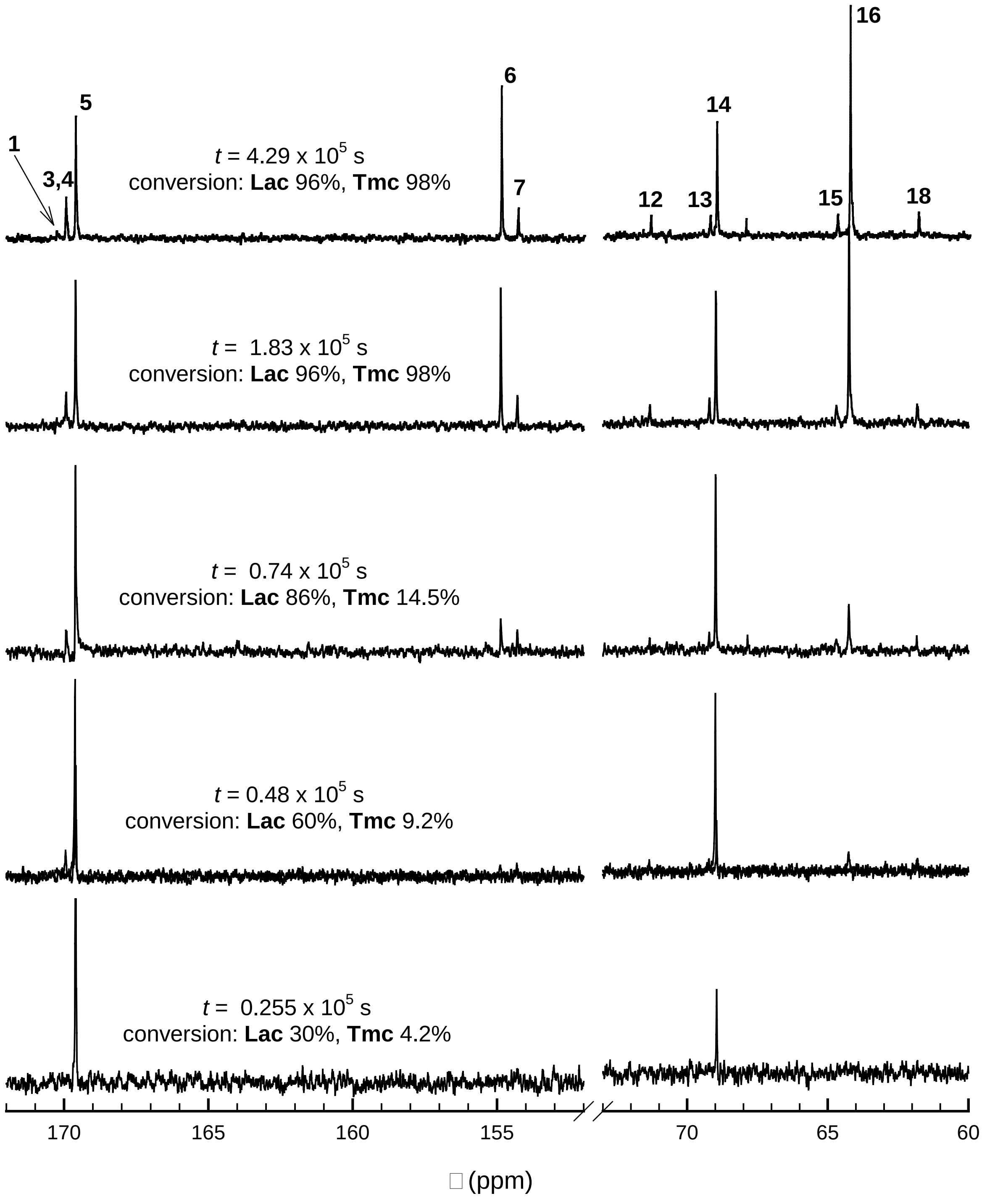

Figure S3. ^{13}C NMR spectrum of copolymer obtained in copolymerization initiated with the mixture of *R*- and *S*-**Ini**. Polymerization Conditions as in Figure S2. Signals are assigned according to shifts given in Table 1 (main tekst).

Figures presenting the simulated distributions of dyads and homoblocks along copolymer chains.

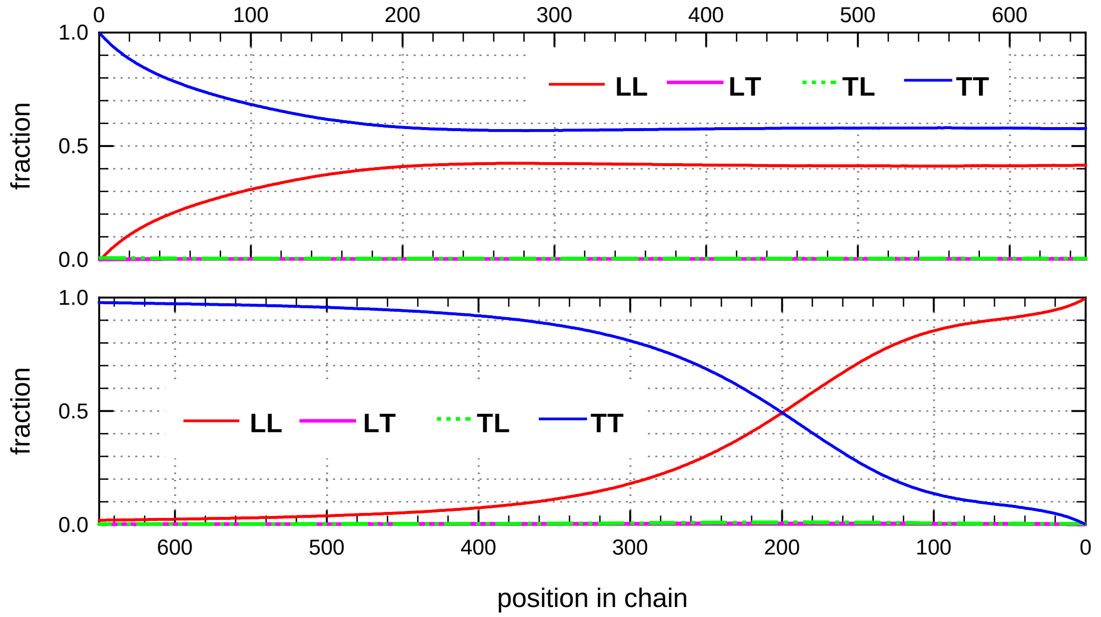

Figure S4. Distribution of copolymer dyads along an average chain expressed as mole fractions for system initiated with *R*-**Ini** (**Tmc*** unimer), calculated for copolymer presented in Figure 6 of the main text. Reactivity ratios: r_L = 21.5 , r_T = 2.5 10^2; z_{LL} = 3.8 10^{-3}, k_{ex}/k_{TT} = 4 10^{-2}; [**Tmc**]$_0$ = 2, [**Lac**]$_0$ = 1.2 mol L^{-1}, and [**Ini**]$_0$ = 2 10^{-3} mol L^{-1} (+ [^{i}PrOH]$_0$ = 4 10^{-3} mol L^{-1} due to *in situ* synthesis of **Ini**). Conversion 95%, DP_n = 507.4, Đ = 1.48. Chain positions counted from chain beginning (top) and chain-end (bottom).

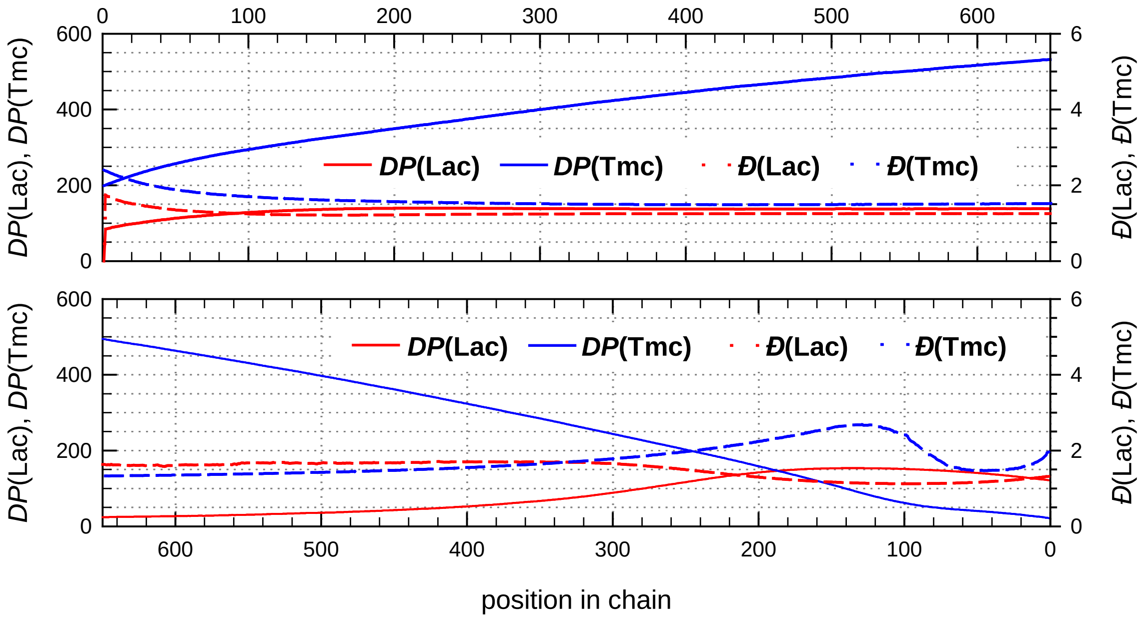

Figure S5. Distribution of homoblock lengths along an average chain for system initiated with *R*-**Ini** (**Tmc*** unimer). For system description see Figure S4. Average homoblock lengths *DP*(M), M = **Lac** or **Tmc**, computed as the number average degrees of polymerization of all homoblocks of a given kind comprising a unit at the given chain position (the block can start before, at, and end after this position). Corresponding block dispersities, Đ(**Lac**) and Đ(**Tmc**), are shown as well.

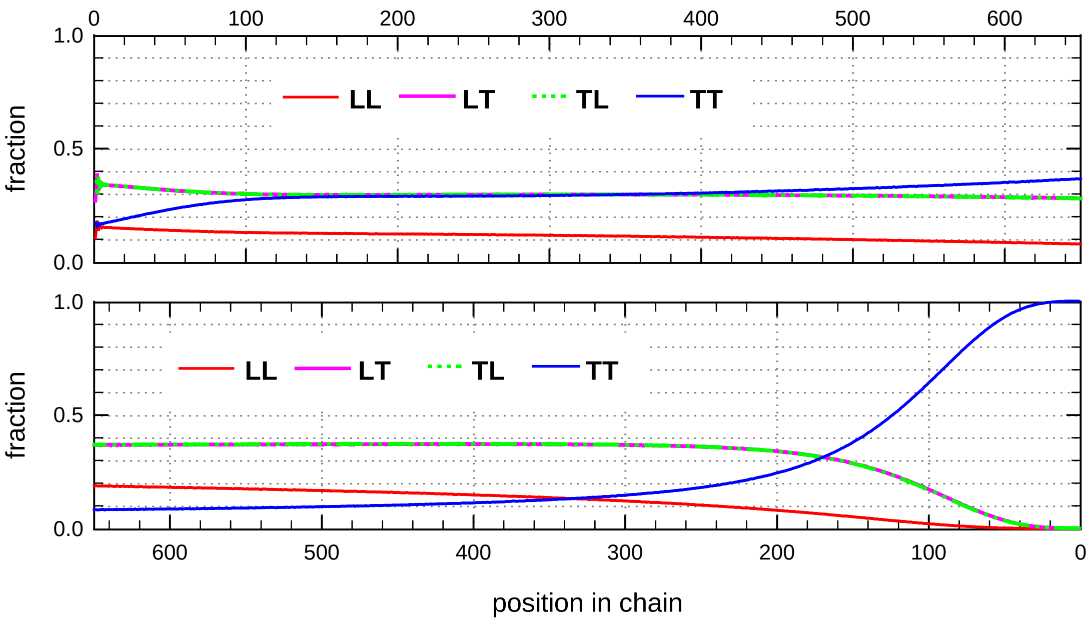

Figure S6. Distribution of dyads along an average chain expressed as mole fractions for system initiated with *S*-**Ini** (mixture of **Lac*** and **Tmc*** unimers), calculated for copolymer presented in Figure 7 of the main text. Reactivity ratios: r_L = 1.11, r_T = 9.7 10^{-2}; k_{ex}/k_{TT} = 8.1; [**Tmc**]$_0$ = 2, [**Lac**]$_0$ = 1.2 mol L^{-1}, and [**Ini**]$_0$ = 2 10^{-3} mol L^{-1} (+ [^{i}PrOH]$_0$ = 4 10^{-3} mol L^{-1} due to *in situ* synthesis of **Ini**). Conversion 95%, DP_n = 506.4, *Đ* = 1.40. Chain positions counted from chain beginning (top) and chain-end (bottom).

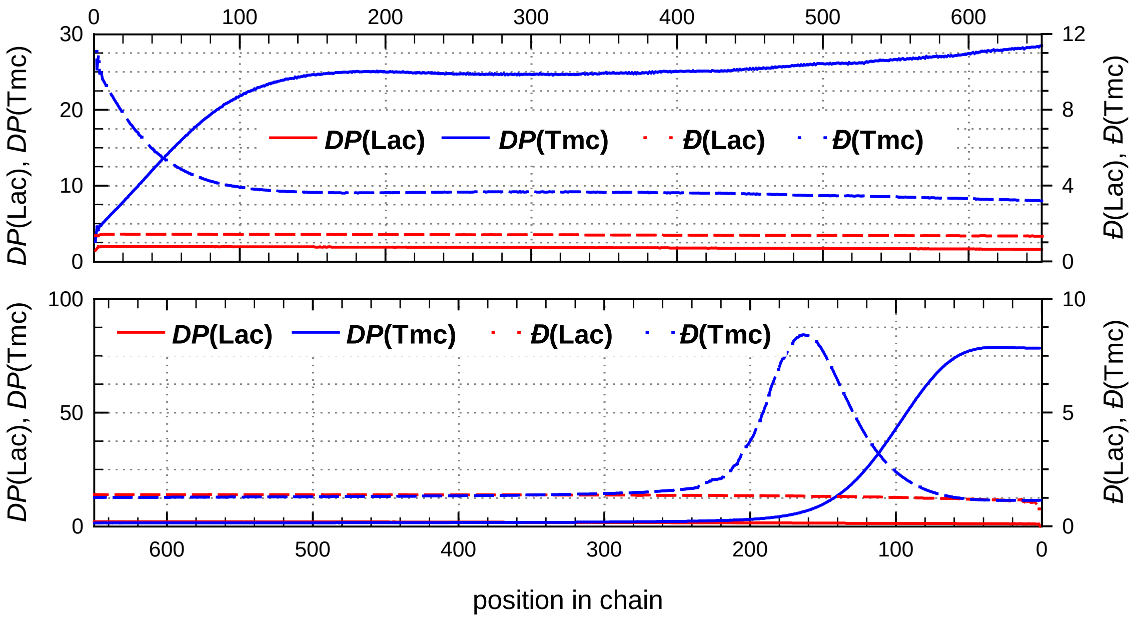

Figure S7. Distribution of homoblock lengths along an average chain for system initiated with *S*-**Ini** (mixture of **Lac*** and **Tmc*** unimers). For system description see Figure S6. Average homoblock lengths *DP*(M), M = **Lac** or **Tmc**, computed as for Figure S5. Corresponding block dispersities, *Đ*(**Lac**) and *Đ*(**Tmc**), are shown as well.

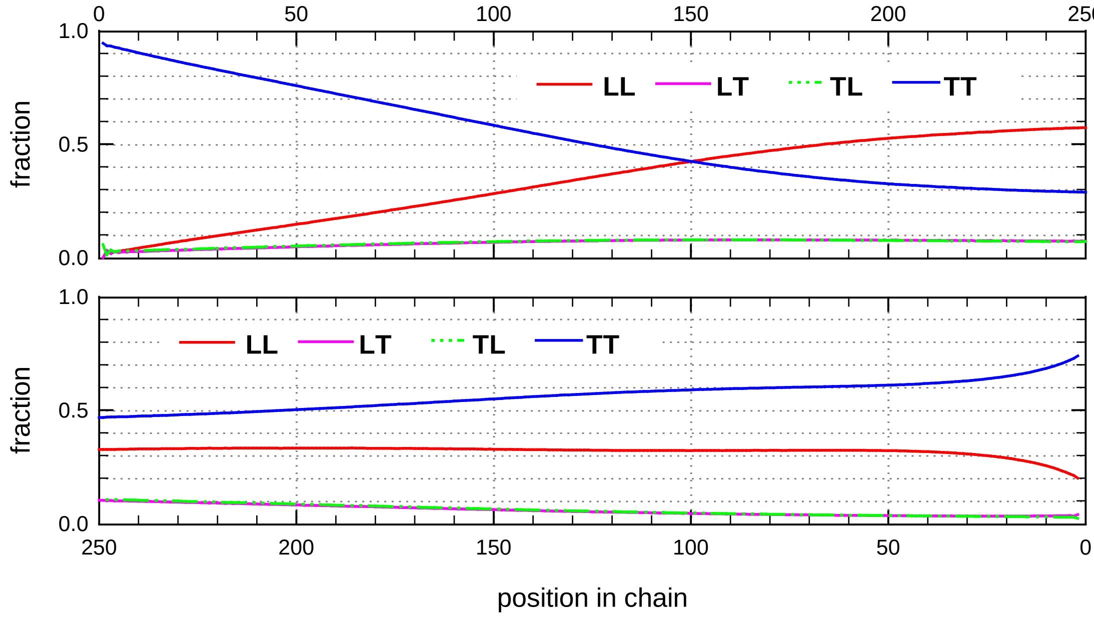

Figure S8. Distribution of dyads along chain length, computed for copolymerization initiated with the mixture of *R*- and *S*-**Ini** (94:6). Conversion 95%, [**Ini**]$_0$ = 5 10^{-2}, [**Lac**]$_0$ = 1.2, [**Tmc**]$_0$ = 2 mol L^{-1}. Reactivity ratios for the corresponding active species as given in Figure S4 and S6, respectively, z_{LLR} = 6.92, z_{LLS} = 1.45, and the ratio of **Tmc** homopropagation rate constants, k_{TTR}/k_{TTS}, being about 0.78. Chain positions numerated from chain beginning (top) and from active center (bottom).

Figures S9-S11 present distributions of homoblocks along chains established using different methods of including blocks in counting their average *DP*.

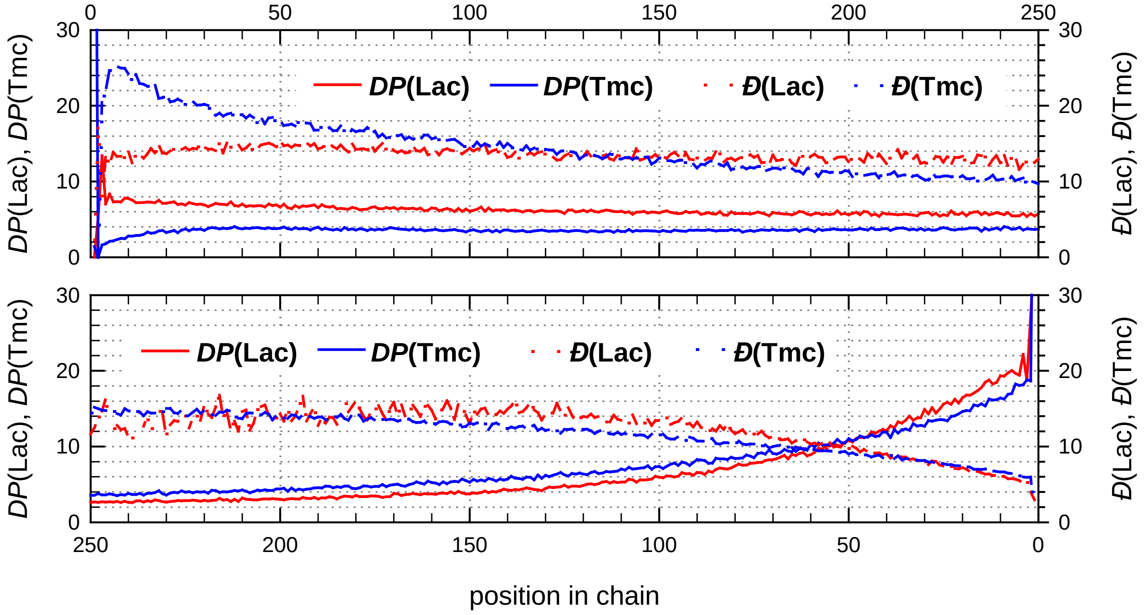

Figure S9. Distribution of homoblocks along chain length, computed for conditions given in Figure S8. Chain positions numerated from chain beginning (top) and from active center (bottom). Average homoblock lengths *DP*(M), M = **Lac** or **Tmc**, are computed as the number average degrees of polymerization of homoblocks of the given kind starting at the considered position. Blocks not starting at this position are disregarded.

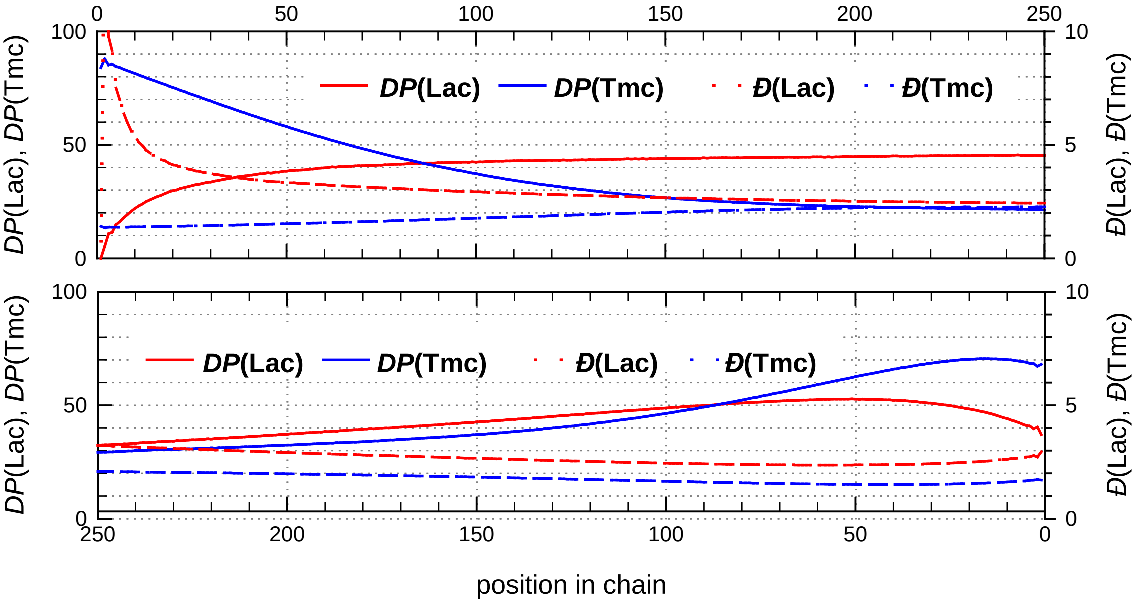

Figure S10. Distribution of homoblocks along chain length, computed for conditions given in Figure S8. Chain positions numerated from chain beginning (top) and from active center (bottom). Average homoblock length *DP*(M), M = **Lac** or **Tmc**, is computed as the number average degree of polymerization of a homoblock of the given kind containing the unit at the given position and its *DP* is counted from this position, disregarding the block part before (closer to chain beginning, top box) or after (closer to chain end, bottom box) this position.

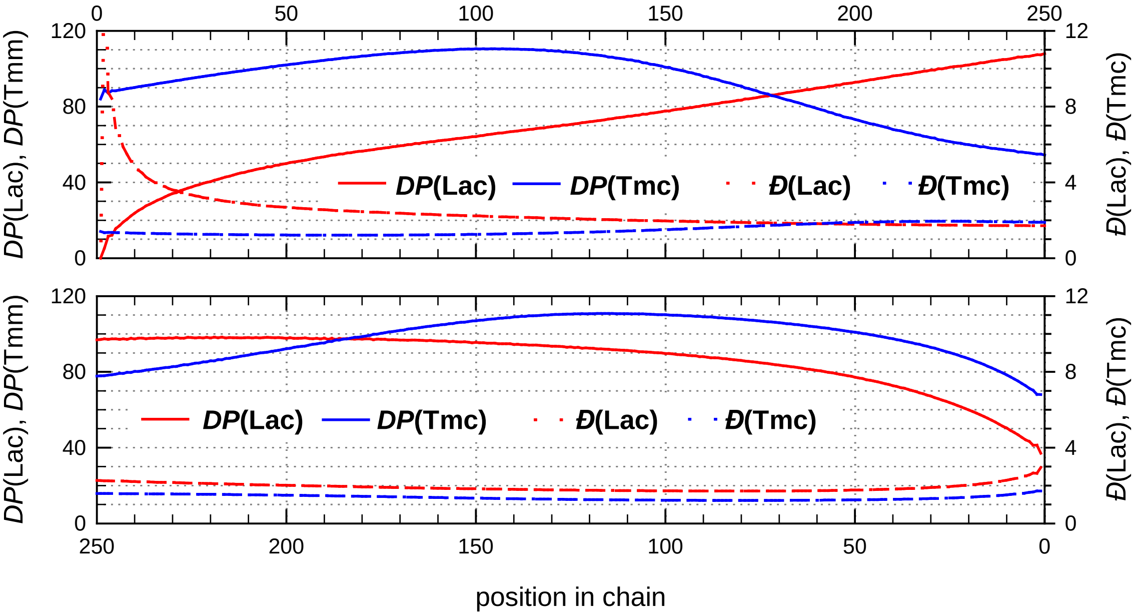

Figure S11. Distribution of homoblocks along chain length, computed for conditions given in Figure S8. Chain positions numerated from chain beginning (top) and from active center (bottom). Average homoblock lengths *DP*(M), M = **Lac** or **Tmc**, are computed as the number average degrees of polymerization of homoblocks of the given kind with a unit at the given chain position, disregarding their first and last unit positions.

One can observe that distribution plots of homoblocks (Figures S9-S11) differ depending on that which blocks are considered in computing their *DP*. Most often in polymer chemistry the block lengths formed at the given reaction time are computed disregarding units formed before the given time. This method of calculating homoblock length corresponds to our method of determining block

lengths starting or continuing from the given position (Figure S10). On the other hand, we think that more informative is considering real lengths of homoblocks containing a unit at the given chain position (Figure S11). However, the overall average chain block lengths are best related with plots shown in Figure S9, where only blocks starting at the given position are regarded. Every copolymer homoblock, while preparing this Figure, is counted only once just like while computing the overall average *DP*. In contrast, while preparing Figures 10 and 11 every homoblock was taken into account for two (in case of dyads) or more chain positions. Consequently, the average block lengths in Figure S9 are much shorter than those in Figure S10 and S11. The average block *DP* in Figure 9 are consistent with the average number of blocks in a chain being about 25, what corresponds to the average homoblock length being about 8.
